# Therapeutic Potential of Metal-Based and PARP Inhibitor Chemotherapy for *BRCA1*-Associated Triple-Negative Breast Cancer

**DOI:** 10.3390/ijms26209881

**Published:** 2025-10-10

**Authors:** Adisorn Ratanaphan

**Affiliations:** Department of Pharmaceutical Chemistry, Faculty of Pharmaceutical Sciences, Prince of Songkla University, Hat-Yai, Songkhla 90112, Thailand; adisorn.r@psu.ac.th; Tel.: +66-7428-8867; Fax: +66-7442-8239

**Keywords:** triple-negative breast cancer, BRCA1 dysfunction, metal-based drugs, PARP inhibitors, synthetic lethality

## Abstract

Triple-negative breast cancer (TNBC) accounts for about 10–15% of all breast cancers and is an aggressive disease with a poor prognosis. There is currently no standard treatment regimen for TNBC patients; thus, chemotherapy remains the main treatment. Anthracycline- and taxane-based regimens are the most widely used in a clinical setting, either alone or in combination with other chemotherapeutic agents, including poly (ADP-ribose) polymerase (PARP) inhibitors and platinum drugs. Platinum drugs have been used particularly in patients with *BRCA1*-mutated TNBC. Preclinical and clinical trials revealed that the response to PARP inhibition was directly correlated to the sensitivity to platinum chemotherapies. Inhibition of PARP enzymes has been shown to specifically target *BRCA1* dysfunctional cells. Therefore, targeting breast cancer cells that possess genetic alterations that are absent in normal cells could be attained by the exploitation of synthetic lethality for the discovery of other candidate metals, i.e., ruthenium-derived compounds, as next-generation drugs for the treatment of TNBC. This prospective approach provides new insight into alternative treatments for breast cancers with *BRCA1*-associated TNBC.

## 1. Introduction

Breast cancer is a diverse histologic disease according to standardized clinicopathologic criteria. In clinical and medical situations, breast cancer has been classified into several biological subtypes with distinct histopathological, genetic, and epigenetic characteristics, clinical outcomes, and dramatically different responses to systemic therapy [1,2,3]. According to molecular subtypes, breast cancer can be categorized as luminal A, luminal B, human epidermal growth factor receptor 2 (HER2) expression, basal-like (BL), and normal breast-like [4]. The BL breast cancer subtype has distinct intrinsic characteristics, including chemotherapeutic responses as well as clinical outcomes [5]. Treatment decisions as well as options of breast cancer are made by the patient and the physician after careful consideration of the suitable treatment available based on subtype, anatomic cancer stage/grade, and biological characteristics of the cancer; patient preferences; and the risks and benefits associated with each treatment protocol. Over the past decades, treatment of breast cancer was considerably uniform, depending on standard protocols such as surgery, radiotherapy, and chemotherapy, with suboptimal outcomes. Surgery is the primary goal to remove the cancer from the breast and to assess the stage of the disease; it is used to treat breast cancer, including lumpectomy, mastectomy, sentinel node biopsy, and axillary lymph node dissection [6]. Radiation therapy using X-rays is recommended to destroy cancer cells remaining in the breast after breast surgery or mastectomy; moreover, it can prevent cancers that have spread to the lymph nodes. Some breast cancers are treated with external or internal radiation (brachytherapy) or both types of therapy [7]. Surgery is often combined with other treatments, including radiation therapy, hormone therapy, chemotherapy, and targeted therapy [8,9,10,11,12]. Hormone therapy is often used to treat breast cancers that are sensitive to hormones. Hormone therapy can be used after surgery or other treatments to decrease the chance of cancer relapse. Chemotherapy refers to the use of anticancer drugs to kill breast cancer cells, and is the most effective when the full dose and cycle of drugs is completed in a timely manner. Several studies support the use of a variety of anticancer agents or regimens. Some of the most common chemotherapy drugs used to treat breast cancer include paclitaxel, docetaxel, doxorubicin, cyclophosphamide, methotrexate, 5-fluorouracil, vincristine, vinblastine, gemcitabine, epirubicin, and platinum drugs [13]. Different chemotherapy drugs exert their actions in different ways and interfere with cancer cells at different phases of their growth and progression. Often called molecular targets, targeted breast cancer therapies are drugs or promising substances that can inhibit the growth and spread of breast cancer cells by interfering with specific molecules involved in cell proliferation.

Triple-negative breast cancer (TNBC) represents the BL subtype overlapping with breast cancer that lacks estrogen receptor (ER), progesterone receptor (PR), and HER2. It is frequently detected in African American or dark-skinned women (15–20%) [14,15,16]. However, not all basal-like cancers are determined via the absence of ER, PR, and HER2, and conversely, not all TNBCs show a basal-like cancer [17]. It has been shown that only two-thirds of triple-negative breast cancers are of the basal-like subtype. The TNBC subtype occurs in 10–15% of all breast cancer cases and is associated with high histological grade, large tumor size, high rates of proliferation with poor prognosis, and high rates of recurrence and metastases within 5 years of the initial diagnosis. TNBC patients have a poorer overall survival than patients with other subtypes of breast cancers, i.e., TNBC patients have a higher risk of the local recurrence rate (LRR) and shorter progression-free survival (PFS) and overall survival (OS) than non-TNBC patients [18,19]. Several lines of evidence have indicated that no expression of ER, PR, and HER2 in conjunction with the expression of high molecular weight basal cytokeratins (CK5/6, 14, and 17), epidermal growth factor receptor (EGFR), proliferating gene marker Ki-67, androgen receptor (AR), cyclin E, and low expression levels of cyclin D1 are commonly found in patients with TNBC [15,20].

TNBC is a highly aggressive subtype of breast cancer with limited targeted treatment options. TNBC has a specific biological profile with many potential molecular targets, including the overexpression of vascular endothelial growth factors (VEGFs) and EGFR, and high rates of BRCA mutation or deficiency in BRCA function (*BRCA*ness). Bevacizumab is the most widely used drug as an anti-VEGF inhibitor with an improvement in PFS for randomized trials of first-line bevacizumab and chemotherapy treatment [21]. Currently, there is emerging evidence on the use of VEGF, EGFR, PARP, and mammalian target of rapamycin (mTOR) inhibitors for the treatment of TNBC. Available prospective randomized data support the use of such targeted agents in TNBC. However, the search for more specific and reliable molecular biomarkers to identify TNBC patients who are more likely to benefit from treatment is ongoing and being evaluated. Several promising therapeutic agents are currently being evaluated for their effectiveness and safety in TNBC. However, the evaluation of therapeutic agents still poses a clinical challenge due to the lack of known targeted agents, guidelines, recognized standards for treatments, and selection of TNBC patients from early prediction of pathologic complete response (pCR) to neoadjuvant chemotherapy. A recent study developed an MRI-based model for predicting complete pathologic response to neoadjuvant chemoimmunotherapy in patients with TNBC [19,22]. Moreover, blood circulating tumor DNA (ctDNA) has been introduced to assess response to treatment and recurrence prediction in patients with early-stage TNBC [15,23,24]. There is currently no single recommended first-line chemotherapy or preferred standard regimen for TNBC treatment. The first-line treatment normally includes a combination of surgery, radiation, and neoadjuvant/adjuvant chemotherapy [25]. TNBC patients have shown high response rates to neoadjuvant chemotherapy. Anthracycline/taxane/platinum chemotherapy-based regimens have been routinely used in the neoadjuvant setting for TNBC; however, anthracycline treatment is associated with undesired cardiotoxicity [26]. Homologous recombination deficiency (HRD) score has been introduced to predict the response to platinum-based neoadjuvant chemotherapy in TNBC patients [27,28]. Retrospective analysis reveals that the combination of paclitaxel with anthracycline may be more advantageous for the treatment of ER-negative and HER2-negative breast cancers. However, the addition of available targeted treatments, including PARP inhibitor (iniparib) and EGFR inhibitor (cetuximab), both in the adjuvant and metastatic settings, unfortunately failed to provide favorable outcomes [29].

Collected data have demonstrated that 20–30% of TNBC patients harbor germline BRCA1 mutations and correlates with decreased BRCA1 mRNA and protein expression [30,31]. In addition, 15–60% of TNBC has been associated with CpG island hypermethylation of the *BRCA1* promoter region that shares similar histological characteristics and clinical outcomes with BRCA1 mutation carriers. It has been reported that *BRCA1* promoter methylation occurs in 32% of TNBC patients and 21% of non-TNBC patients [32,33,34]. In vitro studies showed that a sporadic breast cancer cell, UACC3199, carrying a hypermethylated *BRCA1* promoter, conferred a similar degree of sensitivity to the poly(ADP-ribose) polymerase (PARP) inhibitor, olaparib, as did the mutated *BRCA1* breast cancer cells, MDA-MB-436 and HCC1937, and a combination treatment with carboplatin was more effective than either drug alone [35]. In a similar way, *BRCA1* promoter methylation conferred sensitivity to platinum compounds [36,37]. In recent clinical studies of TNBC patients, *BRCA1* promoter methylation has increased disease-free survival (DFS) and disease-specific survival (DSS) compared with non-TNBC patients who received adjuvant chemotherapy [32].

## 2. Role of BRCA1 in BRCA1-Associated Triple-Negative Breast Cancer

**Figure 1:**
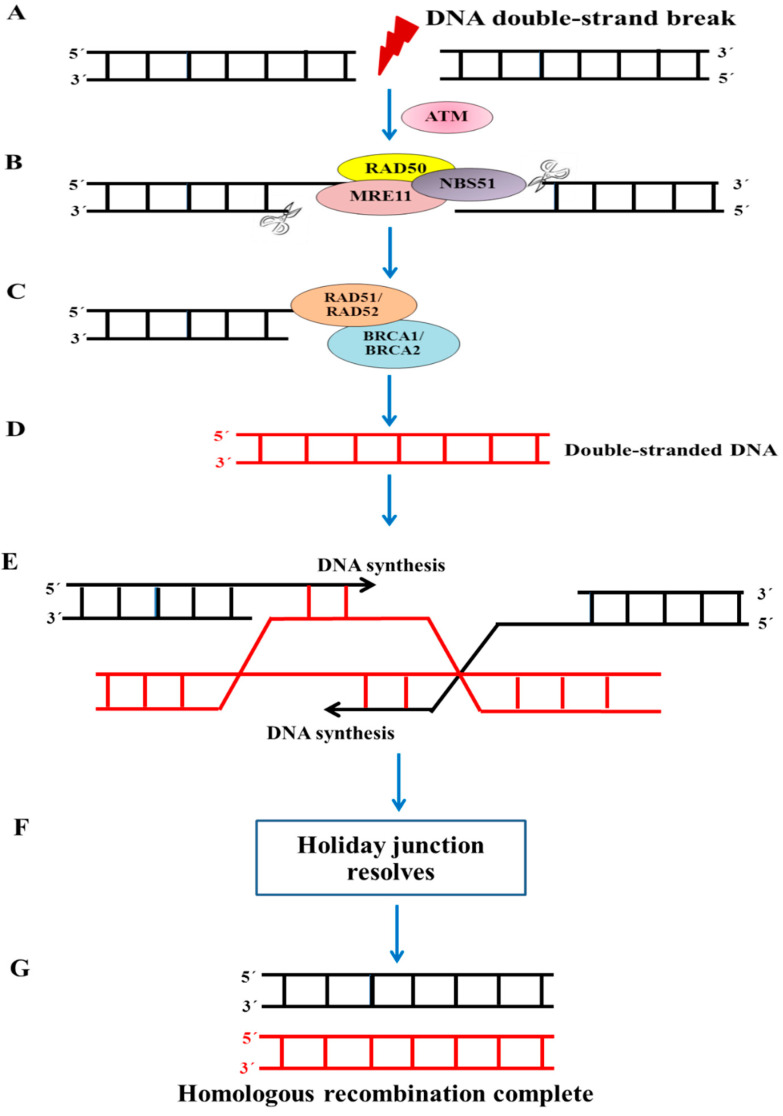
Proposed model for BRCA1-mediated homologous recombination repair.

Since the discovery of the breast cancer susceptibility gene, *BRCA1*, which is located on chromosome 17q, significant advances have been made in understanding the structure and function of the BRCA1 protein. The BRCA1 protein accounts for its ability to maintain genomic integrity and suppress tumor formation through several cellular processes, such as the DNA damage repair pathway, transcription regulation, cell cycle progression, apoptosis, and protein ubiquitination [14,38]. Several lines of evidence have supported an important role of the BRCA1 protein in repairing DNA double-stranded breaks (DSBs) via the homologous recombination (HR) pathway. HR depends on the presence of sister chromatids formed during DNA synthesis as a template for exchanging a homologous sequence with a single-stranded sequence. Thus, this pathway is only available during the S/G2 phases of the cell cycle. Such a repair process requires the recruitment of many proteins to make up a complex that acts as a concert in repairing DNA damage. The underlying molecular mechanism of the HR repair pathway is schematically proposed in Figure 1. A DSB, after being exposed to chemotherapeutics, ionizing radiation, or DNA-damaging agents, activates the ataxia telangiectasia mutated (ATM) kinase, which subsequently catalyzes the MRE11/RAD50/NBS1 (MRN) complex. The MRN complex then recognizes the site of the DNA damage and uses its 5′ ⟶ 3′ exonuclease activity (depicted by scissors) to create single-stranded 3′ ends. Participating proteins such as BRCA1, BRCA2, RAD51, and RAD52 are simultaneously recruited to the DNA-damaged sites [39,40]. BRCA1 interacts with BRCA2 to facilitate the nuclear transport of RAD51. RAD52 aids in RAD51 binding to the damaged ends to form a nucleoprotein filament. RAD51 exchanges a homologous sequence from a single strand within a double-stranded molecule (the sister chromatid as a template) with a single-stranded sequence. Resolvases then restore the junctions (termed Holliday junctions) as a result of homologous recombination. Finally, two copies of an error-free DNA molecule are produced. However, in the absence or impairment of BRCA1, the alternative pathway of non-homologous end joining repair (NHEJ) may be required. The NHEJ pathway is less accurate than the HR pathway because it does not use the sister chromatid as a template [41,42]. This may lead to defective repair and enhance the toxicity of the DNA damage. In addition, BRCA1 has been shown to be involved in nucleotide excision repair (NER), demonstrating a close association between the transcription-coupled repair (TCR) pathway [43,44] and the increased cisplatin sensitivity in *BRCA1*-deficient cells [45,46].

**Figure 2:**
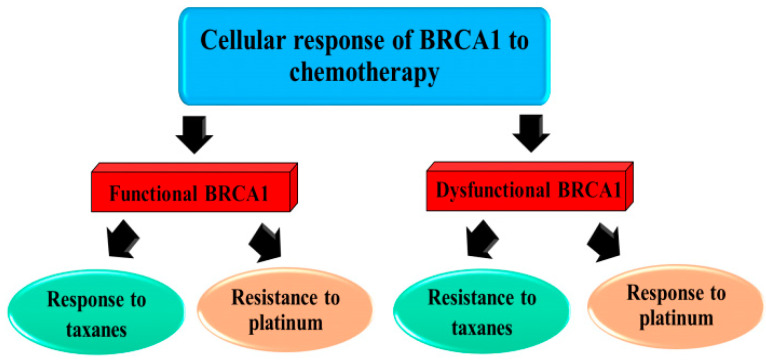
Schematic representation of the potential role of BRCA1 in response to chemotherapy. Dysfunctional BRCA1 results in enhanced sensitivity to platinum-based drugs and reduced response to taxanes.

There is a substantial relationship between dysfunctional BRCA1 and triple-negative status. Breast cancer patients with a BRCA1 mutation are also frequently triple-negative and basal-like [47]. *BRCA1*-associated breast cancers are well categorized as “*BRCA*ness”, which lacks ER/PR/HER2, high-grade, and basal phenotypes. BRCA1 plays an important role in the repair of double-strand breaks (DSBs) through several mechanisms. It is a vital mediator in the HR pathway to repair DSBs (Figure 1). When *BRCA1* is inactivated, breast cancer cells become hypersensitive to DNA damage [25]. If not properly repaired, cancerous cells develop genetic instability and simultaneously enter programmed cell death or apoptosis. Therefore, *BRCA1*-dysfunctional breast cancer may be particularly sensitive to certain classes of DNA-damaging drugs, such as platinum-based drugs as well as their derivatives, and relatively resistant to mitotic spindle poisons, such as taxanes and vinca alkaloids [38]. A decrease in BRCA1 expression was observed in BRCA1-deficient TNBC patients who subsequently became resistant to taxane-based chemotherapy. TNBC patients with non-*BRCA*ness had significantly higher pathological complete responses (pCRs) and a shorter progression-free survival than those with *BRCA*ness tumors. The results indicated that *BRCA*ness breast cancers had a significantly poorer response to taxane regimens than non-*BRCA*ness breast cancers [48]. The neoadjuvant use of cisplatin and carboplatin results in high rates of pCRs in TNBC patients with a defective BRCA1 function [49] (Figure 2).

## 3. Platinum-Based Chemotherapy for BRCA1-Associated Triple-Negative Breast Cancer

**Figure 3:**
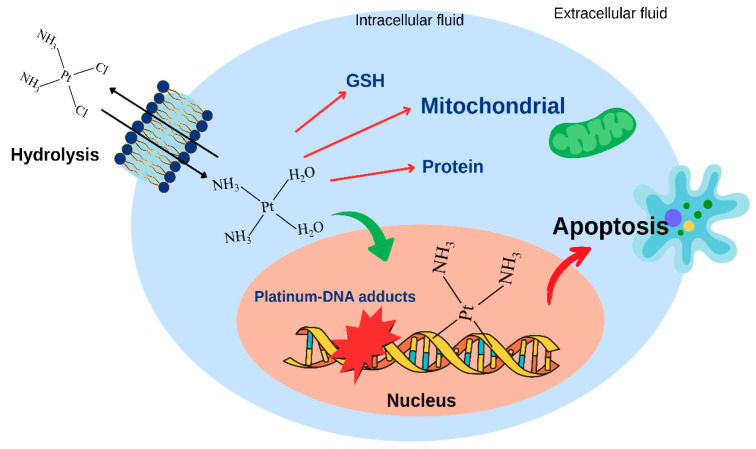
Overview of the mechanism of action of cisplatin.

The discovery of the first platinum complex *cis*-diamminedichloroplatinum(II) as an effective anticancer drug, known as cisplatin, was one of the great success stories in the field of medicinal inorganic chemistry and heralded a new area of anticancer research based on metallopharmaceuticals. The cytotoxicity of cisplatin to cancer cells is directly related to how much drug enters the cell and its cellular accumulation. It is generally accepted that the cytotoxicity of cisplatin results from the interaction of the drug with DNA through the formation of a covalent bond between an activated platinum(II) complex and guanine or adenine in double-stranded DNA. The interaction is apparently preceded by an electrostatic attraction between the positively charged platinum(II) complex and the negatively charged phosphodeoxyribose DNA backbone, and facilitated by a bidirectional diffusion along the DNA backbone [50], followed by the replacement of the remaining chloro ligands before the formation of DNA adducts. The DNA adducts interfere with transcription and translation and lead to apoptosis (Figure 3).

Over the past decades, cisplatin has been widely used in clinical practice for the treatment of a variety of cancers, including testicular, ovarian, head and neck, and lung [51]. Historically, platinum-based chemotherapy has not figured prominently in the treatment of breast cancer. Treatment standards for metastatic breast cancer have included taxane and the use of single-agent capecitabine or vinorelbine for those who relapse shortly after completion of adjuvant taxane treatment. Currently, cisplatin and its derivative carboplatin are among the most effective chemotherapeutic agents in a clinical setting, and are frequently used in combination with other anticancer agents [52] (Table 1). Platinum drugs have been used as a combination regimen of docetaxel and trastuzumab, which is an alternative to anthracycline, taxane, and trastuzumab-based treatments of HER2-positive early breast cancer patients [53]. More experimental neoadjuvant regimens, including platinum drugs combined with taxane, have been shown to have high pCR rates in TNBC patients. Moreover, the role of platinum-based chemotherapy as an alternative therapy to anthracyclines and taxane-resistant breast cancer had an overall response rate of 31%, and a median overall survival of 3–4 months [54]. Therefore, it is necessary to acquire a better understanding of biology and etiology of TNBC, therapeutic targets or molecular biomarkers, as well as patient preferences for the successful treatment of each TNBC subtype.

Despite increasing evidence indicating that *BRCA1*-mutated breast cancer and TNBC confer sensitivity to platinum-derived compounds (Figure 2), randomized data comparing such platinum-based agents versus standard regimens for breast cancer patients in terms of efficacy and safety are still lacking and remain a subject of argument [49,68]. Clinical trials show that carboplatin has a clinical advantage over docetaxel in patients with *BRCA1*-associated TNBC, but not in other TNBC patients. In contrast, recent findings have demonstrated that the application of carboplatin in conjunction with paclitaxel, doxorubicin, and an antiangiogenic drug (bevacizumab) did not improve pCR rates in patients harboring HER2-positive or *BRCA1*-associated TNBC [68]. The association between *BRCA1* deleterious somatic mutation status and response to a taxane/carboplatin neoadjuvant chemotherapy in women with TNBC has been investigated, with the results showing a higher pCR rate than non-carriers (60% vs. 30%, *p* = 0.32). Therefore, breast cancer patients with *BRCA1* somatic mutations are more likely to respond to the platinum drug carboplatin [27,69]. Nonetheless, a controversial finding showed that there is no advantage of neoadjuvant cisplatin (pCR rate of 18%) over an anthracycline (doxorubicin) and cyclophosphamide (AC-based regimen, pCR rate of 26%), after surgery, in patients with HER2-negative, *BRCA1*-associated breast cancer, whether HER-positive, HER-negative, or TNBC [68]. Consequently, the decision on chemotherapeutic treatment should be carefully considered to avoid any other adverse drug reactions (ADRs) or events. To meet criteria, the application of poly(ADP-ribose) polymerase (PARP) inhibitors alone or in combination with chemotherapeutic drugs/agents seems to be an attractive neoadjuvant option for patients carrying these breast cancer subtypes. The results of a Phase II randomized trial of cisplatin in the presence/absence of veliparib in metastatic TNBC and/or germline BRCA-associated breast cancer (Swog S1416) demonstrated superior progression-free survival (PFS) with PARP inhibitor monotherapy compared with chemotherapy for gBRCA mutation [57].

## 4. PARP Inhibitors for BRCA1-Associated Triple-Negative Breast Cancer

One of the bottlenecks in cancer-related drug discovery is the identification of tumor-selective characteristics. Normally, cancer cells often have DNA repair defects. This leads to the conceptual framework that a combinative inhibition within DNA repair pathways in cancer cells may result in synthetic lethality. Therefore, several investigators have exploited this idea to gain a better understanding of the concept of loss-of-function mutations found in DNA repair genes. In eukaryotic cells, there are more than 100 genes that repair DNA damage [70]. A large body of evidence has demonstrated that HR-defective cancers are highly sensitive to DNA-damaging agents, cytotoxic drugs, and PARP inhibitors [71,72]. In addition, the DSB repair defect in *BRCA1*-mutated cells has been exploited in a synthetically lethal approach with PARP inhibition [52,73]. Moreover, *BRCA1*-mutated breast cancers have been shown to be more immunogenic than HR-proficient cancers.

**Figure 4:**
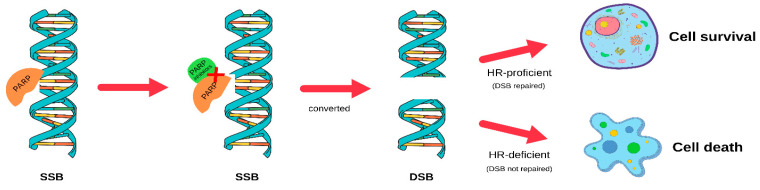
Schematic representation of the mechanism of PARP inhibitors.

**Figure 5:**
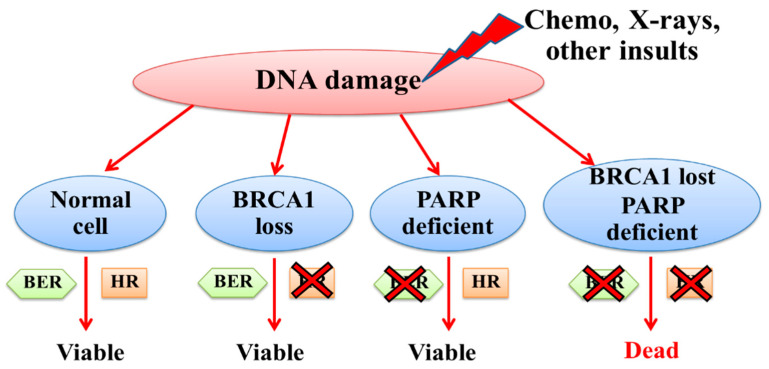
Schematic representation of the synthetic lethality of cancer cells with defective *BRCA1*.

PARP is a highly abundant DNA-binding protein that plays an important role in estrogen-related transcription, chromatin remodeling, and the DNA repair pathway, particularly in the base excision repair of single-stranded DNA breaks (SSBs) [3,74]. Inhibition of PARP leads to the accumulation of SSBs that finally cause the formation of DSBs (Figure 4) after interfering with DNA replication processes. These DSBs cannot be accurately repaired in cancer cells with HR deficiency [75]. Therefore, the inhibition of PARP using synthetic killing agents could be advanced as a novel targeted therapy for breast cancer patients with dysfunctional BRCA1 (Figure 5) [76].

Several PARP inhibitors (PARPis) have been developed with different antitumor activity [77,78,79]. It is well known that they exhibit synthetic lethal effects on tumors defective in the *BRCA1* gene, which encodes the protein required for efficient HR repair [80]. Cells harboring *BRCA1* mutations are much more sensitive to PARP inhibitors than wild-type cells [73]. As previously mentioned, triple-negative breast cancer patients are closely associated with defective *BRCA1*. PARP inhibitors impair BER through PARP inhibition by trapping the PARP complex to DNA, subsequently causing DSBs [81]. BRCA mutations have been validated as biomarkers for patient selection for PARP inhibitors in clinical settings. PARPis have been developed as single-agent treatments for BRCA1/2-deficient breast, ovarian, and prostate cancers [82,83]. Knockout of the PARP genes in experimental cell models impaired normal embryonic development and led to high levels of SSBs, eventually resulting in the accumulation of DSBs, cell cycle arrest, and/or cell death [84]. Many PARPis have progressed to clinical trials, including olaparib, talazoparib, veliparib, rucaparib, niraparib, mefuparib hydrochloride, pamiparib, and suraparib [17,85,86,87]. FDA approved the PARP inhibitors olaparib and talazoparib in 2018/2019 for clinical treatment of refractory metastatic breast cancer with deleterious germline *BRCA1/2* mutations, *BRCA*-mutated HER2-negative metastatic breast cancer, gBRCAm metastatic pancreatic cancer, and maintenance of *BRCA*-mutated (gBRCAm or sBRCAm) advanced epithelial ovarian cancers [88,89]. In addition, the trial demonstrated a positive correlation between higher HRD scores and better response to talazoparib, particularly in patients with PALB2 mutations [89]. Nonetheless, in germline BRCA1/2 (gBRCA1/2)-mutated HER2-negative advanced breast cancer, talazoparib did not significantly improve overall survival over chemotherapy [90]. Compared with other PARP inhibitors, pamiparib showed improved penetration across the blood–brain barrier in mice [86,91]. Recently, a potent PARP inhibitor, senaparib, has been developed and shown to be highly potent in cell viability tests against tumor cells with BRCA1/2 mutations [76]. However, acquired resistance to PARP inhibitors limits their clinical efficacy [92]. Novel PARP inhibitors with increased PARP trapping capacity, such as veliparib derivatives, have been developed as promising candidates [93]. In addition, it has been reported that the emergence of acquired resistance caused by secondary genetic or epigenetic modification that restores HR repair frequently occurs [94,95,96].

## 5. Cellular Resistance to Platinum Drugs

**Figure 6:**
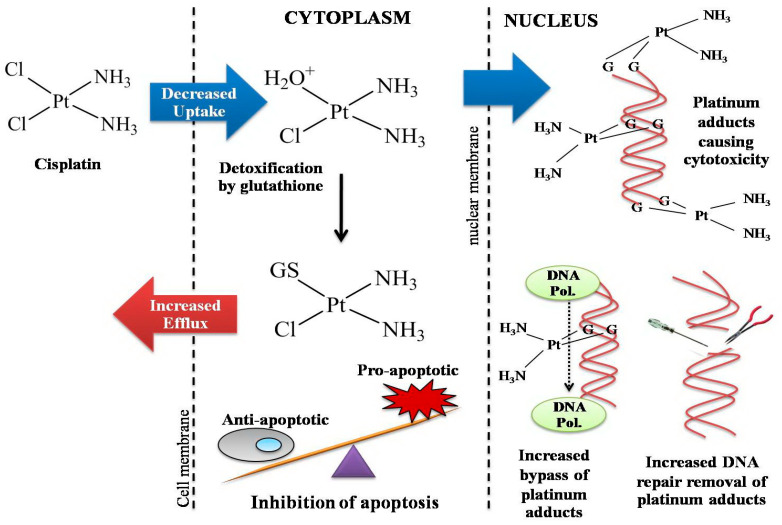
Schematic representation of cellular resistance to cisplatin.

Presently, there is concern about the clinical limitations of the anticancer platinum drugs, cisplatin and its derivatives, that are widely used as chemotherapeutic drugs for the treatment of TNBC. The clinical disadvantages of cisplatin include severe toxicity, such as nephrotoxicity and neurotoxicity; its limited applicability to a narrow range of tumors; and limited efficacy for cancer cells that have developed platinum resistance [97,98]. Clinical data have shown that breast cancer patients at first responded well to platinum drugs; however, patients became drug-resistant after prolonged treatment. Cellular resistance to cisplatin is caused by a wide variety of mechanisms, including decreased drug accumulation, increased levels of the intracellular thiols, and increased DNA repair [99] as depicted in Figure 6.

### 5.1. Decreased Drug Accumulation in Cisplatin Resistance

Decreased drug accumulation could be attributed to decreased influx, decreased intracellular binding, or increased efflux. The mechanism of cisplatin uptake is believed to occur through a combination of passive diffusion and carrier-mediated transport processes [100]. Several copper transport proteins are found to play significant roles in drug transport and drug resistance [101,102]. The high-affinity copper transporter CTR1 has been implicated in cisplatin transport in non-renal cells. Evidence derived from cell models has indicated that CTR1 regulates cisplatin cytotoxicity by interfering with drug uptake. The copper exporters ATP7A and ATP7B have been found to be involved in cisplatin resistance by enhancing efflux [103,104]. A recent study showed that Cox17, a copper chaperone that delivers cuprous ions to mitochondria for the activation of cytochrome c oxidase, can transport platinum to mitochondria. An increased Cox17 level potentiates platinum accumulation in mitochondria and enhances the cytotoxicity of cisplatin. Moreover, dysfunctional Cox17 was found to reduce cisplatin sensitivity of the cell [105,106].

### 5.2. Increased Binding to Intracellular Thiol Molecules

Cisplatin resistance has resulted from an increase in the intracellular level of glutathione (GSH) [107]. GSH is a tripeptide thiol, γ-glutamylcysteinylglycine, and the major cellular non-protein thiol with high concentrations (0.5–10 mM) in cells. It plays an important role in a variety of physiological functions in cellular defense and metabolism, including the protection of cells from oxidative stress and the detoxification of electrophilic compounds [108]. Intracellular GSH can regulate the amount of platinum being transported. An increase in GSH can lead to a decrease in copper concentration, which enhances the expression of CTR1 [103]. The binding of cisplatin to GSH is usually considered to be associated with drug resistance due to the reduced DNA platination [109]. Elevated GSH levels are observed in some cisplatin-resistant cancer cells. The GSH–platinum complex is eliminated from cancer cells using an ATP-dependent glutathione S-conjugate export pump [108], which results in a reduction in the intracellular accumulation of the platinum complex. In addition, overexpression of metallothionein (MT) has been consistently observed in cisplatin-resistant tumor cell lines. These findings indicate that cisplatin resistance may be prevented or reduced through the regulation of metallothionein synthesis.

### 5.3. Increased DNA Repair

There are several DNA repair pathways involved in the repair of DNA damage caused by various anticancer drugs, including the platinum drug cisplatin [110]. Each DNA repair pathway can increase repair activity in cancer cells, enabling them to survive DNA damage that is induced by chemotherapeutic treatments, which results in resistance to chemotherapeutic drugs [111]. Although many patients initially respond favorably to cisplatin-based chemotherapy. Such resistance is often driven by enhanced DNA repair capacity, as evidenced by decreased platinum adduct accumulation, increased repair synthesis, and reactivation of cisplatin-damaged plasmids.

Cisplatin–DNA adducts can be excised and repaired before replication or DNA synthesis via base excision repair (BER) or nucleotide excision repair (NER). BER excises a single damaged DNA base or a short strand harboring the damaged base [112], while NER excises a single-stranded DNA molecule (24–32 base pairs) containing the DNA lesions [113]. Preclinical-derived data have shown that the NER pathway is required for the majority of intrastrand cisplatin–DNA adducts [114]. In addition, the excision repair cross-complementing 1 (*ERCC1*) gene, a key factor in NER, has been shown to undergo transcriptional upregulation in certain patients following cisplatin treatment. Collectively, these results underscore the importance of gene-level DNA repair mechanisms as critical determinants of cisplatin resistance.

The overexpression of breast cancer suppressor protein 1 (BRCA1)-mediated HR was observed in cisplatin-resistant breast and ovarian carcinoma cell lines [115,116] as well as the occurrence of secondary *BRCA1* mutations in breast cancers previously treated with platinum drugs [117]. The tumor suppressor gene *p53* has also been linked to DNA repair capacity. Disruption of *p53* in human breast cancer cells increases cisplatin sensitivity, likely due to impaired DNA repair ability. Moreover, inhibition of the BRCA/Fanconi anemia pathway sensitizes tumor cells to cisplatin, whereas deficiencies in this pathway confer cisplatin resistance. Furthermore, activation of proto-oncogenes *c-fos* and *c-myc* enhances downstream gene expression and stimulates repair protein activity, thereby further strengthening cellular DNA repair capacity [118].

### 5.4. Epigenetics in Resistance to Cisplatin

Epigenetics, including DNA methylation, histone modifications, and microRNA silencing, play a pivotal role in the development of cisplatin resistance. Aberrant hypermethylation of CpG islands within tumor suppressor gene promoters disrupts key cellular processes such as cell cycle, DNA repair, apoptosis, and carcinogen metabolism, thereby facilitating cancer progression [119]. Recent studies have reported that TNBC patients with germline *BRCA1* mutation and *BRCA1* promoter methylation become resistant to platinum-based therapy [120,121]. In addition, epigenetic remodeling mechanisms are responsible for DNA damage response (DDR) dysregulation and hence drug resistance. The emergence of acquired resistance caused by secondary genetic or epigenetic modification that restores HR repair frequently occurs [122].

However, no clear-cut dominant mechanism of resistance to cisplatin has been identified in human cancer cells. Cisplatin resistance of a particular cell may be due to several possible mechanisms. Moreover, a given cell may use more than one mechanism or can simultaneously develop several mechanisms of resistance. Other mechanisms may be involved in cellular resistance to cisplatin, such as altered folate metabolism [123], changes in oncogene expression [118], modulation by protein kinases [124], and loss of DNA mismatch repair [125]. In addition, an increased tolerance to DNA damage can be another explanation for increased resistance to cisplatin. Cells can achieve damage tolerance by bypassing DNA lesions during replication or transcription [126].

## 6. Cellular Resistance to PARP Inhibitors

**Figure 7:**
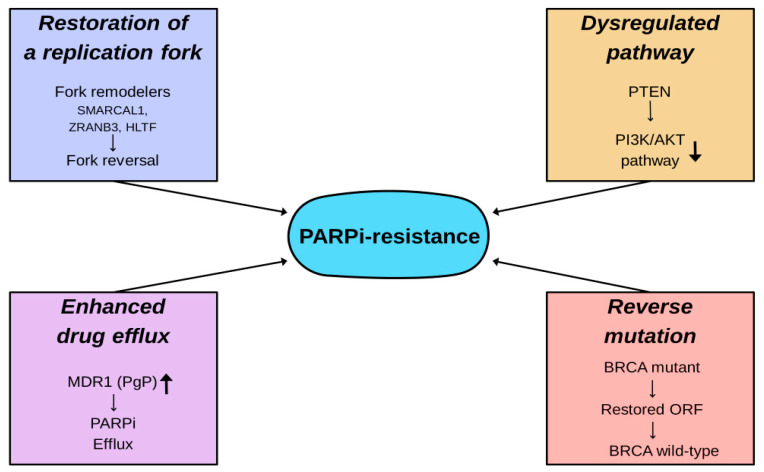
Cellular resistance to PARP inhibitors.

Cellular resistance to PARP inhibitors is caused by a wide variety of mechanisms, including reverse mutation, restoration of replication fork stability, dysregulation within molecular signaling pathways, and enhanced drug efflux, as depicted in Figure 7.

### 6.1. Reverse Mutation

PARP inhibitor resistance is closely associated with the reversion of BRCA1/2 mutations, which restore the open reading frame (ORF) and are, therefore, capable of the synthesis of native proteins. This reversion mutation reduces the synthetic lethality induced by PARP inhibition, ultimately leading to PARP inhibitor resistance [127]. Moreover, HR deficiency has been shown to activate the non-homologous end joining (NHEJ) pathway. NHEJ is a major pathway in the G1 phase of the cell cycle that directly connects both ends of truncated DNA double-strands and is, therefore, error-prone. In contrast, proliferating cells most likely employ HR, an error-free pathway that utilizes the sister chromatid as a template and is particularly activated during the late S and G2 phases of the cell cycle. While HR is active during the S and G2 phases of the cell cycle, NHEJ functions during interphase. In HR-defective cells, treatment with PARP inhibitors induces synthetic lethality. When NHEJ remains functional, cells still undergo cell death upon PARP inhibition. In contrast, when NHEJ is blocked, HR-defective cells can escape synthetic lethality, thereby contributing to the development of resistance to PARP inhibitors [128].

### 6.2. Restoration of Replication Fork Stability

*BRCA*ness cancer cells exhibit HR deficiency and undergo replication fork collapse mediated by MRE11, which underlies their strong cytotoxic response to PARP inhibitors [129]. Replication fork remodelers such as SMARCAL1, ZRANB3, and HLTF can partially counteract this vulnerability by promoting fork reversal, recruiting protective factors, stabilizing forks, preventing excessive DNA damage accumulation, and allowing cancer cell survival despite PARP inhibitor treatment. This fork remodeling represents a critical mechanism of acquired PARP inhibitor resistance in *BRCA1* mutant cancers, playing a role irrespective of HR restoration [130].

### 6.3. Dysregulation Within Molecular Signaling Pathways

Poly-ADP-ribosylation (PARylation) is a post-translational modification characterized by the addition of ADP-ribose units to substrate proteins. This process is catalyzed by the poly(ADP-ribose) polymerase (PARP) family of ADP-ribosyl transferases and exerts multiple functions in cells, including proliferation, differentiation, and DNA repair [131]. PARylation requires the binding of PARP1 or PARP2 to DNA. If this binding is disrupted, the cytotoxic effects of PARP inhibitors are reduced, leading to PARP inhibitor resistance and the failure to achieve synthetic lethality [132]. It has been implicated that the PTEN protein (phosphatase and tensin homolog) and the PI3K/AKT signaling pathways are involved in PARP inhibitor resistance [133]. Loss of PTEN activity enhances PI3K/AKT signaling, interferes with DSB repair, and promotes the growth of *BRCA1*-deficient cancer cells by upregulating BRCA1 expression [134]. PTEN-deficient cancer cells have been closely associated with decreased RAD51 expression, one of the key proteins involved in HR [135], thereby rendering cancer cells sensitive to PARP inhibitors. In contrast, PTEN-proficient cancer cells can contribute to PARP inhibitor resistance [136].

### 6.4. Enhanced Drug Efflux

Cellular resistance through enhanced drug efflux is mediated primarily by ATP-binding cassette (ABC) transporters. Overexpression of Multidrug-Resistance Protein 1 (MDR1)/P-glycoprotein (PgP, encoded by Abcb1a/b) has been observed in *BRCA*-mutated breast and ovarian cancer cells, leading to a reduced intracellular concentration of PARP inhibitors, and consequently, PARP inhibitor resistance [137,138]. Inhibition of MDR1/PgP with agents such as tariquidar, verapamil, or elacridar has been shown to reverse resistance to PARP inhibitors [139,140].

In addition, it has been reported that cellular resistance to platinum drugs and PARP inhibitors in *BRCA1*-defective cancer cells is caused by overexpression of a microRNA(miR-622)-mediated regulation of NHEJ [141]. Moreover, the really interesting gene (RING) domain-deficient BRCA1 proteins (Rdd-BRCA1) are capable of contributing to PARP inhibitor and platinum resistance [142]. Therefore, to overcome resistance to platinum drugs and PARP inhibitors, prospective possibilities including the discovery of next-generation Pt/PARP selective inhibitors, novel drug delivery carriers, as well as an antibody–drug conjugation system (ADC) could be alternative approaches to address the drawbacks of chemotherapy and meet medical requirements [84,94,143,144,145,146].

## 7. Ruthenium-Based Chemotherapy for BRCA1-Associated Triple-Negative Breast Cancer

**Figure 8:**
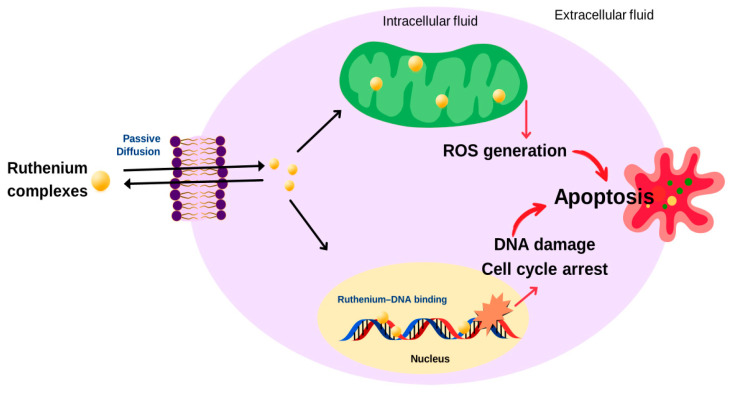
Mechanism of action of ruthenium complexes.

**Figure 9:**
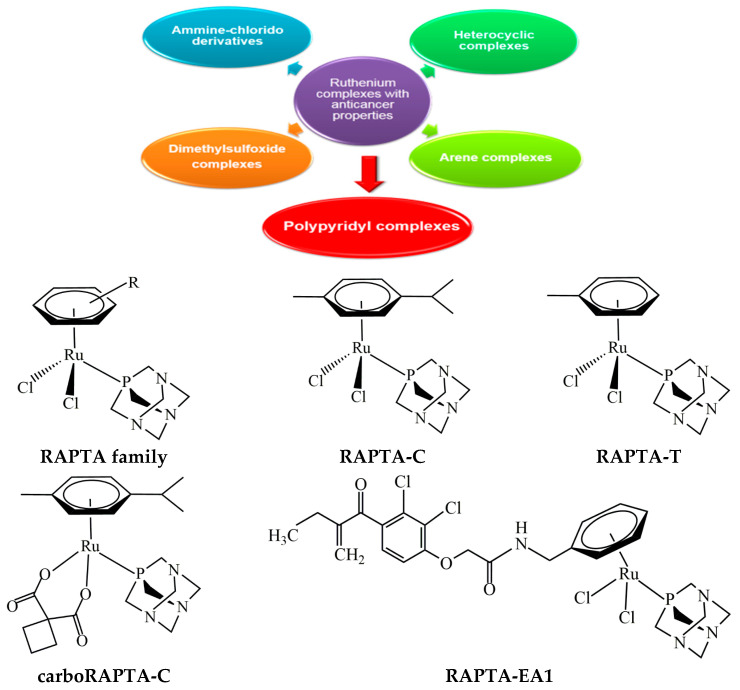
Classification of ruthenium complexes with anticancer properties and structures of RAPTA-type complexes.

A search for non-platinum metal-based chemotherapeutics has been extensively investigated with recent advances in the development of new anticancer agents [147,148,149,150,151,152,153,154,155,156,157,158,159,160,161,162,163,164,165,166,167,168,169,170,171,172,173,174,175,176]. Among non-platinum-based compounds, a number of ruthenium-centered complexes have received much attention as promising alternatives to conventional anticancer platinum-based drugs. These complexes can form DNA adducts and induce crosslinking, such as effective anticancer platinum compounds, but often with significantly lower general toxicity. The lower toxicity of ruthenium complexes is partly due to a function of the ligands attached to the metal center, and it is believed to be a function of the metal itself. Ruthenium can adopt different oxidation states under physiological conditions [147,148], and the more active Ru(II) oxidation state is promoted in the hypoxic environment of cancer cells, whereas the more inert Ru(III) state is promoted in healthy tissues. Moreover, some ruthenium complexes can mimic iron (Fe) in binding to important carrier proteins such as transferrin [149,150,151,152,153], which is postulated to be a specific delivery mechanism to rapidly dividing cells, including tumor cells, because of their higher iron requirement. Ruthenium complexes appear to exhibit different mechanisms of action compared with platinum compounds (Figure 8). They can interact with DNA and disrupt replication and transcription processes. They also interfere with telomerase activity by binding to G-quadruplex structures of telomeric DNA and inhibit topoisomerases, ultimately inducing programmed cell death. These properties of ruthenium complexes give rise to less toxicity than the approved cisplatin or any other anticancer platinum(II) complexes, and may be able to overcome drug resistance that develops in the current platinum-based treatments. Moreover, they may also target cancer types that are not sensitive to existing drugs. A number of ruthenium complexes with a variety of ligands have been shown to display promising anticancer properties [154,155,156,157,171,172,173,174,175,176,177,178,179,180,181,182,183,184,185]. Several ruthenium complexes have already been shown to exhibit excellent in vivo antitumor activity, and some complexes have even entered clinical trials [150,151,177,178,179,180] (Figure 9, Table 2).

Dyson et al. developed the ruthenium-based complexes, called the RAPTA family or Ru(*η*^6^-arene)(PTA)Cl_2_ (PTA = 1,3,5-triaza-7-phosphaadamantane) [155,171,175]. A monodentate ligand, PTA, exhibits remarkable water solubility and thermal stability and forms stable metal complexes. Due to its unique properties, PTA-containing ruthenium complexes have been shown to be promising antimetastatic agents [154,172,182,183,184]. The biochemical mode of action of the RAPTA derivatives is different from that of anticancer platinum-based drugs on cancer cells [149,152,155,171]. It is generally accepted that cellular DNA is the main target for classical platinum drugs, but intracellular proteins, including thiol-containing proteins, are evidenced for the RAPTA compounds [181,182,183]. This class of ruthenium compounds shows high affinity to the thiol moiety of cysteine residues of proteins that play important roles in DNA regulation, histone modification, signal transduction, cell growth, and epigenetic pathways [181,182,183]. Despite the lower affinity for DNA, several RAPTA complexes display excellent in vivo activity, reducing the number and weight of solid metastases, but not affecting the primary tumor. The prototype compound, [Ru((*η*^6^-*p*-cymene)Cl_2_(pta)], termed RAPTA-C (Figure 5), remains the best characterized compound of the series, and the underlying molecular mechanism has been demonstrated to involve mitochondrial-induced apoptosis [157]. RAPTA-C exhibited its IC_50_ value of >300 μM for both normal and cancer cell lines. As such, it would not be considered a cytotoxic agent. In addition, RAPTA-C exhibited broad-acting antitumor efficacy with intrinsic angiostatic activity. The drug combination between RAPTA-C and the epidermal growth factor receptor (EGFR) inhibitor, erlotinib, resulted in strong synergistic inhibition of cell viability in human endothelial and human ovarian carcinoma cells [184,185]. Moreover, RAPTA-C accumulated on cellular chromatin, potentially forming adducts at specific histone sites on the nucleosome core [182]. This implied that RAPTA-C primarily targets proteins that are becoming increasingly implicated as relevant to the mode of action of RAPTA compounds.

Previous studies have demonstrated that RAPTA complexes regulate the marker genes involved in the apoptotic pathway, cell cycle progression, and the expression of BRCA1 [154,156,173,186,187]. RAPTA-EA1 (a RAPTA compound with ethacrynic acid (EA) tethered to the arene ring) and Ru(*η*^6^-toluene)(pta)Cl_2_, termed RAPTA-T, significantly decreased *BRCA1* replication in *BRCA1*-defective HCC1937 cells compared with *BRCA1*-proficient MCF-7 cells [188]. Conversely, *BRCA1* mRNA expression was apparently upregulated in HCC1937 cells in the presence of the ruthenium compound, whereas it was downregulated in MCF-7 cells. However, such a ruthenium compound caused a reduction in BRCA1 expression in the tested breast cancer cells. In this case, *BRCA1* mRNA expression was inversely proportional to its protein expression, as it was in HCC1937 cells. This negative correlation may derive from diverse subtypes of breast cancer [4]. It was implied that downregulation of *BRCA1* mRNA expression in sporadic *BRCA1-proficient* MCF-7 cells might be due to ruthenation of the *BRCA1* promoter region that may interfere with the transcription factors required for *BRCA1* transcription activity and finally lead to decreased mRNA expression [154]. In contrast, in HCC1937 cells, whose *BRCA1* mRNA expression was increased while having a significantly reduced replication, this might result from more favorable ruthenation within the *BRCA1* structural gene, meanwhile removing the Ru-*BRCA1* adducts. The resulting data have revealed a differential cellular response for *BRCA1*-deficient and *BRCA1*-proficient breast cancer cells to the compound. Moreover, RAPTA compounds have been found to interact with the *N*-terminal region of the BRCA1 RING domain proteins, both wild-type and clinically relevant variant proteins (D67E and D67Y) [189]. The binding of the compounds to BRCA1 proteins led to the release of Zn^2+^ ions in a dose- and time-dependent manner; moreover, changes in thermal events eventually resulted in the impaired function of the RING heterodimer BRCA1/BARD1-mediated E3 ubiquitin ligase activity that played an important role in DNA damage response [45,46]. The D67Y variant exhibited a decrease in ubiquitination function and was more susceptible to RAPTA treatment than the other variants tested. Moreover, treatment of the BRCA1 protein with the ruthenium compound in combination with the PARP inhibitor olaparib resulted in 5-fold inhibition of the E3 ligase activity, indicating a synergism [189]. Likewise, metallo-intercalator ruthenium(II) polypyridyl complexes caused the inactivation of BRCA1-mediated E3 ubiquitin ligase activity [47]. These findings indicate that the zinc finger motif of dysfunctional BRCA1 proteins could be a molecular target for ruthenium-based agents in the treatment of breast cancer.

## 8. Synergistic Effects of Olaparib in Combination with Platinum/Ruthenium-Based Anticancer Agents in BRCA1-Associated Triple-Negative Breast Cancers

The rational combination of the metal-based agents with PARP inhibitors has long been hypothesized and developed to achieve synergistic activity [65,190,191,192,193,194,195,196,197,198,199,200]. A combination treatment between platinum-containing drugs and a PARP inhibitor is one of the therapeutic strategies to improve their efficacy in *BRCA1/2*-deficient breast cancer cells [26,64,65,66,67]. Preclinical and clinical trials revealed that the response to PARP inhibition was directly correlated to sensitivity to platinum chemotherapies [49,52,55,58,72,73]. The assessment for olaparib in combination with chemotherapy in patients with advanced ovarian, breast, and other solid tumors has shown encouraging efficacy [60,61,62]. The excellent sensitivity of these cancers to olaparib, either single or combined treatment with the platinum-containing drugs, has provided strong support for using olaparib in combination with promising metal-based drugs as novel targeted therapeutics against *BRCA*-deficient cancers. With the positive outcome on progression-free survival, olaparib has been further evaluated in Phase III clinical trials in combination with cisplatin/carboplatin with gemcitabine in *BRCA1*-associated and triple-negative breast cancers [65]. However, it has been demonstrated that neoadjuvant olaparib did not improve any pCR rates, PFS, or OS when added to carboplatin–paclitaxel and anthracycline-based chemotherapy in TNBC patients with wild-type *BRCA1/2* [65]. This lethality is a possible explanation because the cancer cells with defects in the *BRCA1/2* gene are defective in HR repair. Nonetheless, resistance to PARP inhibitors or platinum chemotherapy in *BRCA1* mutant metastatic breast cancer has been found [65,196]. Additionally, a recent study revealed that olaparib combined with cisplatin may exert its synergistic action on breast cancer through platinum drug resistance and the longevity regulating pathway, and downregulate the expression of targeted genes involved in apoptotic and DNA metabolic pathways [197,198]. RAPTA derivatives have been shown to be significantly more effective against *BRCA1*-deficient breast cancer cells than cisplatin [52,187,188]. Moreover, the combination treatment of RAPTA with olaparib exhibited a synergistic effect in *BRCA1*-deficient HCC1937 (5382insC mutation) cell line, in a dose-dependent manner [52]. Drug sensitivity in BRCA1-mutated cells might likely be related to a defective BRCA1 that is incapable of repairing DNA damage induced by the ruthenium compound or olaparib treatment, ultimately leading to cell death. Moreover, olaparib can be exploited for other types of cancer, regardless of *BRCA1* status. Synergism has been observed for triple-negative *BRCA1*-proficient breast cancer cells treated with anticancer organometallic compounds and other promising inhibitors [199]. Recently, the combination of DNA-binding ruthenium(II) polypyridyl complexes and olaparib showed synergy in triple-negative *BRCA1*-proficient breast cancer cells [192,199]. However, a ruthenium-derived glutathione transferase (GSTP-1) inhibitor, combined with olaparib, dramatically reduced the expression of the BRCA1 protein as well as the inhibition of *BRCA1* replication in triple-negative *BRCA1* wild-type MDA-MB-231 breast cancer cells [52]. It appears that drug susceptibility in triple-negative *BRCA1*-proficient breast cancer cells is linked to the reduced expression of the BRCA1 protein in repairing DSBs after ruthenium exposure; meanwhile, olaparib targets the PARP enzyme, which leads to cancer cell death via synthetic lethality.

Although preclinical and clinical trials have shown that PARP inhibitors are effective for *BRCA1*-associated cancers, some limitations to the synthetic lethality approach still exist, such as evidence for therapeutic efficacy, side effects, and resistance to PARP inhibitors. Not all patients carrying *BRCA1*-deficient breast cancer responded equally well; some patients experienced relevant side effects from PARP inhibitors, including myelosuppression, gastrointestinal toxicity, hypercholesterolemia, fatigue, and teratogenicity [200,201]. Moreover, side effects from platinum drugs included nephrotoxicity, neurotoxicity, ototoxicity, myelosuppression, and damage to normal tissue for prolonged treatment [202] (Table 3 and Table 4). In addition, it has been shown that tumors develop resistance to PARP inhibitors by a variety of mechanisms, such as upregulation of the proteins involved in drug efflux pumps, hypomorphic activity of mutant *BRCA1* alleles, and rewiring of the DNA damage response [196]. Moreover, cancer cells can survive or share some characteristics by using a salvage DNA repair pathway because DNA repair pathways can overlap, although this is not well defined, to overcome a defect in a single pathway.

## 9. Future Perspectives

A major drawback of anticancer agents is their lack of selectivity for tumor tissues, including breast cancer. This causes systemic toxicity, severe side effects, and gives rise to poor response rates. Accordingly, increasing the therapeutic index of the drug while minimizing systemic toxicity would be the best strategy to improve breast cancer therapy. It is well established that the characteristics of breast cancerous tissue are quite different from normal tissues; for instance, breast cancerous tissue is characterized by a lack of a functional lymphatic drainage system and more permeable and leakier blood vessels. However, differential responses to chemotherapy among patients with breast cancer become relevant, as well as the development of drug resistance. Hence, it is crucial to assess the responses of early breast cancer patients to a given chemotherapeutic agent without the presence of adverse drug effects. Combining PARP inhibitors with ruthenium-based agents to induce *BRCA*ness or inactivate the BRCA1 protein via its HR deficiency not only represents a potential approach for TNBC treatment but also a challenging strategy that remains largely unexplored [217]. In addition, other molecularly targeted biomarker proteins beyond BRCA mutations, including HR-related (ATM, BARD1, CHEK2, MRE11, NBS51, RAD50, RAD51, PALB2, FANCA, 53PB1, CDC25), cell cycle/apoptosis signaling pathways (CHKs, CDKs, PD-L1), replication fork stability (ATR/WEE1), hypoxia-induced signaling (HIF-1), cellular stresses/oxidative stresses (p38 MAPK, NRF2, NAD^+^/NADH-SIRT3 axis), and therapeutic approaches for TNBC without germline *BRCA* mutations are encouraging to achieve preclinical and clinical outcome assessments [218,219,220,221,222,223,224]. To the best of our knowledge, the responsiveness of TNBC to targeted therapy is significantly influenced by the expression of molecularly predictive biomarkers and hormone receptor status. Therefore, precision medicine in combination with suitable drug delivery as a tool for tailoring therapeutic strategies is required to improve both preclinical and clinical outcomes. In addition, a better understanding of the precise mechanisms of action of the anticancer metal-based candidates and their potential synergy with existing therapeutic drugs could pave the way for the development of novel metal-centered drugs that overcome treatment resistance and improve efficacy and selectivity.

## Figures and Tables

**Table 1:** Platinum drugs/PARP inhibitors as single and combination therapy for the treatment of TNBC.

Chemotherapy	TNBC (n)	Setting	Outcomes	References
Cisplatin	86	Metastatic	RR 37%	[55]
Olaparib	27	Metastatic	pCR 41%	[56]
Cisplatin/veliparib	162	Neoadjuvant	pCR 74%	[57]
Cisplatin/docetaxel	27	Metastatic	ORR 59%	[58]
Carboplatin/docetaxel	28	Neoadjuvant	pCR 86%	[59]
Carboplatin/veliparib	634	Neoadjuvant	pCR 47%	[60]
Carboplatin/veliparib	107	Neoadjuvant	pCR 61%	[61]
Gemcitabine/carboplatin/iniparib	258	Metastatic	ORR 34%	[62]
Paclitaxel/carboplatin	24	Neoadjuvant	pCR 33%	[63]
Paclitaxel/doxorubicin/cyclophosphamide/carboplatin	60	Neoadjuvant	pCR 51%	[64]
Eribulin/carboplatin	22	Neoadjuvant	pCR 40%	[26]
Paclitaxel/carboplatin/olaparib	559	Neoadjuvant	pCR 51%	[65]
Mitomycin C/vinblastine/cisplatin	34	Metastatic	ORR 41%	[66]
Gemcitabine /carboplatin/iniparib	80	Neoadjuvant	ORR 36%	[67]

pCR, pathological complete response; ORR, overall response rate; RR, response rate; and CR, complete response.

**Table 2:** Preclinical/clinical studies and relevant anticancer activity of ruthenium complexes.

Ruthenium Complexes	Phase/ Status	Mechanism of Action/Clinical Challenges	Ref.
NAMI-A	Phase II	- Inhibits the formation of new blood vessels - Inhibits adhesion, migration, and α5β1 integrin - Strong inhibition of tumor malignancy and metastasis; however, clinical development was limited by patient side effects	[177,178]
KP-1019	Phase I	- Modulates intracellular ROS levels - Induces apoptosis through mitochondria or the MAPK/P38 pathway, and blocks the cell cycle in the G2/M phase - Enters Phase I for colorectal tumors, but clinical development was limited due to low solubility	[177,178,179]
KP-1339	Phase I	- Modulates intracellular ROS levels - Induction of apoptosis through mitochondria or the MAPK/P38 pathway, and blocks the cell cycle in the G2/M phase - Early clinical trials	[177,178]
TLD1433	Phase Ib	- Light activation, generating cytotoxic singlet oxygen and radical oxygen species, leading to cell death - Currently in phase trials for the treatment of non-muscle invasive bladder cancer	[177,178,180]
BOLD-100	Phase I	- Currently in Phase II trials for advanced gastrointestinal cancer	[177,178,180]
RM175	Preclinical	- Successful in vitro and in vivo assessments with IC50 values similar to cisplatin in vitro - Being studied in advanced clinical trials	[177]
RAED-C	Preclinical	- Targets the DNA of chromatin - Activity is similar to cisplatin (DNA-targeting proclivity and apoptosis profile) - Cytotoxicity against various cancer cell lines, including cisplatin-resistant cells - Highly active in the primary tumor - Being studied in advanced clinical trials	[177,181,182,183]
RAPTA-C	Preclinical	- Mono-aquated form is the most abundant species - Multitargeted anticancer activity with antimetastatic properties and induced pH-dependent DNA damage - Exhibits broad acting antitumor efficacy with intrinsic angiostatic activity - Suppresses cathepsin B and thioredoxin reductase activities - Modifies proteins and histone–DNA interactions - The steric bulk of the phosphaadamantane ligand is the primary factor that distinguishes histone/DNA site selectivity - Being studied in advanced clinical trials	[177,181,182,183,184]

**Table 3:** Mechanism, toxicity, and efficacy of platinum complexes, PARP inhibitors, and ruthenium complexes on TNBC.

Class	Primary Mechanism	Toxicity	Efficacy
**Platinum complexes**	Platinum complexes exert cytotoxicity by forming DNA crosslinks that disrupt replication and transcription, leading to the accumulation of unrepaired DNA lesions, cell cycle arrest, and apoptosis in TNBC [203]	- Dose-limiting - Nephrotoxicity - Neurotoxicity - Ototoxicity - Myelosuppression - Damage to normal tissues if used for a long time [202]	- Increase the pathologic complete response (pCR) rate in TNBC when combined with chemotherapy in the neoadjuvant setting [204]
**PARP inhibitors**	PARP inhibitors indicate antitumor effects in TNBC by blocking the repair of single-strand DNA breaks (SSBs), leading to the accumulation of double-strand breaks (DSBs) during replication. In *BRCA1/2* mutant or HR-deficient TNBC, these lesions cannot be effectively repaired, resulting in synthetic lethality [205]	- Myelosuppression - Gastrointestinal toxicities - Fatigue - Hypercholesterolemia - Teratogenicity [201]	- Strong efficacy in BRCA-mutated TNBC [205]
**Ruthenium complexes**	Ru(III) complexes are involved in TNBC by accumulating in mitochondria, causing mitochondrial dysfunction, ROS generation, and membrane depolarization, which leads to DNA damage and cell death. Additionally, ruthenium inhibits the protein expression of macrophage colony-stimulating factor (M-CSF), which is relevant to the PI3K/AKT/mTOR pathway, thereby reducing migration, invasion, and angiogenesis of cancer cells [181]	- Ru(II) complexes induce toxicity in TNBC through multiple mechanisms depending on the ligands - Several Ru(II) complexes exhibit low IC50 values in TNBC, which typically shows poor response to current drugs [206]	- Ru(II) complexes can be designed with various ligands, including traditional drugs or bioactive ligands, to achieve more effective treatment of TNBC [206]

**Table 4:** Comparison of the mechanism, advantages, limitations, and clinical status of platinum drugs, PARP inhibitors, and ruthenium complexes.

Class	Mechanism	Advantages	Limitations	Clinical Status
**Platinum** **drugs**	The cytotoxicity of platinum complexes arises from the covalent binding of platinum atoms to the N7 position of purine bases in DNA, forming platinum adducts that generate intrastrand and interstrand crosslinks. This blocks replication and transcription, inhibits DNA synthesis, induces cell cycle arrest, and ultimately triggers apoptosis in cancer cells [207]	Widely used for the treatment of cancer - Ovarian cancer - Lung cancer - Gastrointestinal cancer - Germ-cell cancer and various other malignancies [208]	High toxicity - Nephrotoxicity - Ototoxicity - Neurotoxicity Drug resistant [207]	- Cisplatin FDA approved (1978) - Carboplatin FDA approved (1989) - Oxaliplatin FDA approved (2002) - Nedaplatin Not FDA approved; approved in Japan (1995) - Heptaplatin Not FDA approved; approved in South Korea (1999) - Lobaplatin Not FDA approved; approved in China (2010) - Satraplatin Not FDA approved [208,209]
**PARP** **inhibitors**	PARP inhibitors induce synthetic lethality by inhibiting PARP1/2 catalytic activity, preventing the repair of single-strand DNA breaks (SSBs) and leading to their accumulation. This results in the formation of double-strand breaks (DSBs). In HR-deficient tumors (e.g., BRCA1/2 mutants), DSBs cannot be efficiently repaired, ultimately leading to cell death (apoptosis) [95]	- Extended progression-free survival (PFS) in ovarian and breast cancer [210] - Highly effective in HR-deficient tumors and BRCA1/2 mutation [207] - Exhibited promising clinical activity against various solid tumors [211]	- Drug resistance caused by HRR restoration (e.g., BRCA1/2 reversion mutations, loss of PARP-1 binding, or PARP-1 mutations) [212] - Limited efficacy in tumors without BRCA1/2 mutations [211] - Development of therapy-related myeloid neoplasms (t-MNs) [213]	- Olaparib FDA approved (2014) - Rucaparib FDA approved (2016) - Niraparib FDA approved (2017) - Talazoparib FDA approved (2018) [214]
**Ruthenium complexes**	Ru(II) complexes inhibit tumor growth and metastasis by entering the nucleus, binding to DNA, inducing DNA damage, and causing cell cycle arrest. Additionally, ruthenium can localize to mitochondria, leading to mitochondrial dysfunction, increased ROS generation, and apoptosis of cancer cells [215]	- High redox potential, allowing Ru(II) complexes to selectively damage target tumor cells [177] - Administered via multiple routes (e.g., oral, intravenous, and intraperitoneal) [177] - Exhibited tumor cell selectivity with minor effects on healthy cells [177] - Mimicked the iron-binding properties of serum albumin and transferrin [177] - Enters cells via transferrin receptors, inducing apoptosis [176] - Several oxidation states (e.g., Ru(II), Ru(III), and Ru(IV)) under physiological conditions [176] - Pro-drug potential: the inactive Ru3+ state in circulation can be reduced to the active Ru2+ state within target cells [178] - Slow ligand exchange kinetics [178] - Less toxic than platinum-based drugs [216] and capable of overcoming platinum-drug resistance [178]	- The anticancer mechanism of Ru(II) complexes remains poorly understood [179] - Clinical challenges persist; for example, NAMI-A remains in Phase I trials due to patient adverse effects [177]	Ru(II) complexes are not FDA-approved and are still under clinical evaluation in humans - KP-1019 (Phase I) - KP-1339 (Phase I) - NAMI-A (Phase II) - TLD1433 (Phase Ib) - BOLD-100 (Phase I) - RM175, RAED-C, and RAPTA-C remain in preclinical studies [177,178]

## Data Availability

Data are available within the article. Reasonable inquiries for additional information can be directed to the corresponding author.

## References

[1] Sahin T.K., Rizzo A., Guven D.C., Aksoy S. (2025). Post-progression treatment options after CDK4/6 inhibitors in hormone receptor-positive, HER2-negative metastatic breast cancer. Cancer Treat. Rev..

[2] Huppert L.A., Gumusay O., Idossa D., Rugo H.S. (2023). Systemic therapy for hormone receptor positive/human epidermal growth factor receptor 2-negative early stage and metastatic breast cancer. CA Cancer J. Clin..

[3] Rajan A., Nadhan R., Latha N.R., Krishnan N., Warrier A.V., Srinivas P. (2021). Deregulated estrogen receptor signaling and DNA damage response in breast tumorigenesis. Biochim. Biophys. Acta Rev. Cancer.

[4] Perou C.M., Sorlie T., Eisen M.B., van de Rijn M., Jeffrey S.S., Rees C.A., Pollack J.R., Ross D.T., Johnsen H., Akslen L.A. (2000). Molecular portraits of human breast tumours. Nature.

[5] Badve S., Dabbs D.J., Schnitt S.J., Baehner F.L., Decker T., Eusebi V., Fox S.B., Ichihara S., Jacquemier J., Lakhani S.R. (2011). Basal-like and triple-negative breast cancers: A critical review with an emphasis on the implications for pathologists and oncologists. Mod. Pathol..

[6] Veronesi U., Cascinelli N., Mariani L., Greco M., Saccozzi R., Luini A., Aguilar M., Marubini E. (2002). Twenty-year follow-up of a randomized study comparing breast-conserving surgery with radical mastectomy for early breast cancer. N. Engl. J. Med..

[7] Whelan T.J., Pignol J.-P., Levine M.N., Julian J.A., MacKenzie R., Parpia S., Shelley W., Grimard L., Bowen J., Lukka H. (2010). Long-term results of hypofractionated radiation therapy for breast cancer. N. Engl. J. Med..

[8] Jhaveri K.L., Neven P., Casalnuovo M.L., Kim S.-B., Tokunaga E., Aftimos P., Saura C., O’Shaughnessy J., Harbeck N., Carey L.A. (2025). Imlunestrant with or without Abemaciclib in Advanced Breast Cancer. N. Engl. J. Med..

[9] Bardia A., Cortés J., Bidard F.-C., Neven P., Garcia-Sáenz J., Aftimos P., O’Shaughnessy J., Lu J., Tonini G., Scartoni S. (2024). Elacestrant in ER+, HER2- Metastatic Breast Cancer with ESR1-Mutated Tumors: Subgroup Analyses from the Phase III EMERALD Trial by Prior Duration of Endocrine Therapy plus CDK4/6 Inhibitor and in Clinical Subgroups. Clin. Cancer Res..

[10] Bidard F.-C., Kaklamani V.G., Neven P., Streich G., Montero A.J., Forget F., Mouret-Reynier M.-A., Sohn J.H., Taylor D., Harnden K.K. (2022). Elacestrant (oral selective estrogen receptor degrader) versus standard endocrine therapy for estrogen receptor-positive, human epidermal growth factor receptor 2-negative advanced breast cancer: Results from the randomized phase III EMERALD trial. J. Clin. Oncol..

[11] Shah M., Lingam H., Gao X., Gittleman H., Fiero M.H., Krol D., Biel N., Ricks T.K., Fu W., Hamed S. (2024). US Food and Drug Administration approval summary: Elacestrant for estrogen receptor-positive, human epidermal growth factor receptor 2-negative, ESR1-mutated advanced or metastatic breast cancer. J. Clin. Oncol..

[12] Chaubal R., Talker E., Chitra J., Kadam R., Gardi N., Ursekar R., Kadam A., Singh A., Sale S., Pandey S. (2025). Genomic landscape of hormone therapy-resistant HR-positive, HER2-negative breast cancer. Breast Cancer Res. Treat..

[13] Mason S.R.E., Willson M.L., Egger S.J., Beith J., Dear R.F., Goodwin A. (2023). Platinum-based chemotherapy for early triple-negative breast cancer. Cochrane Database Syst. Rev..

[14] Ratanaphan A. (2012). A DNA repair BRCA1 estrogen receptor and targeted therapy in breast cancer. Int. J. Mol. Sci..

[15] Sabit H., Abouelnour S., Hassen B.M., Magdy S., Yasser A., Wadan A.-H.S., Abdel-Ghany S., Radwan F., Alqosaibi A.I., Hafiz H. (2025). Anticancer Potential of Prebiotics: Targeting Estrogen Receptors and PI3K/AKT/mTOR in Breast Cancer. Biomedicines.

[16] Coakley M., Villacampa G., Sritharan P., Swift C., Dunne K., Kilburn L., Goddard K., Pipinikas C., Rojas P., Emmett W. (2024). Comparison of circulating tumor DNA assays for molecular residual disease detection in early-stage triple-negative breast cancer. Clin. Cancer Res..

[17] Vagia E., Mahalingam D., Cristofanilli M. (2020). The Landscape of Targeted Therapies in TNBC. Cancers.

[18] Rakha E.A., El-Sayed M.E., Green A.R., Lee A.H.S., Robertson J.F., Ellis I.O. (2007). Prognostic markers in triple-negative breast cancer. Cancer.

[19] Zagami P., Carey L.A. (2022). Triple negative breast cancer: Pitfalls and progress. NPJ Breast Cancer.

[20] Yip H.Y.K., Papa A. (2021). Signaling Pathways in Cancer: Therapeutic Targets Combinatorial Treatments, and New Developments. Cells.

[21] O’Shaughnessy J., Romieu G., Dieras V., Byrtek M., Duenne A.A., Miles D. (2010). Abstract P6-12-03: Meta-Analysis of Patients with Triple-Negative Breast Cancer (TNBC) from Three Randomized Trials of First-Line Bevacizumab (BV) and Chemotherapy Treatment for Metastatic Breast Cancer (MBC). Cancer Res..

[22] Liu B., Wu L., Liu C., Long X., Hu S., Zhang L., Liu Z., Liang C. (2025). Baseline and Early Treatment MRI Model for Predicting Complete Pathologic Response to Neoadjuvant Chemoimmunotherapy in Patients With Triple-Negative Breast Cancer. AJR Am. J. Roentgenol..

[23] Pascual J., Attard G., Bidard F.-C., Curigliano G., De Mattos-Arruda L., Diehn M., Italiano A., Lindberg J., Merker J.D., Montagut C. (2022). ESMO recommendations on the use of circulating tumour DNA assays for patients with cancer: A report from the ESMO Precision Medicine Working Group. Ann. Oncol..

[24] Alkassis S., Suresh Y., Lipsyc-Sharf M., Zhang S., Gianni C., Medford A., Bardia A., Ashouri S., Kapoor N. (2025). Circulating tumor DNA detection of local recurrence in a patient with early stage triple-negative breast cancer. Breast Cancer Res. Treat..

[25] Zhang L., Chen Y., Cheng M.-Y., Zhuang X., Zou J., Wei D., Lin Y.-Y., Zhang Y., Wang K. (2022). Homologous recombination deficiency predicts the response to platinum-based neoadjuvant chemotherapy in early-stage triple-negative breast cancer patients: A systematic review and meta-analysis. Ther. Adv. Med. Oncol..

[26] Masuda N., Bando H., Yamanaka T., Kadoya T., Takahashi M., Nagai S.E., Ohtani S., Aruga T., Suzuki E., Kikawa Y. (2021). Eribulin-based neoadjuvant chemotherapy for triple-negative breast cancer patients stratified by homologous recombination deficiency status: A multicenter randomized phase II clinical trial. Breast Cancer Res. Treat..

[27] Yuan Y., Lee J.S., Yost S.E., Li S.M., Frankel P.H., Ruel C., Schmolze D., Robinson K., Tang A., Martinez N. (2021). Phase II Trial of Neoadjuvant Carboplatin and Nab-Paclitaxel in Patients with Triple-Negative Breast Cancer. Oncologist.

[28] Telli M.L., Hellyer J., Audeh W., Jensen K.C., Bose S., Timms K.M., Gutin A., Abkevich V., Peterson R.N., Neff C. (2018). Homologous Recombination Deficiency (Hrd) Status Predicts Response to Standard Neoadjuvant Chemotherapy in Patients with Triple-Negative or Brca1/2 Mutation-Associated Breast Cancer. Breast Cancer Res. Treat..

[29] Baselga J., Stemmer S., Pego A., Schneeweiss A. (2011). Abstract PD01-01: Cetuximab + cisplatin in estrogen receptor-negative, progesterone receptor-negative, HER2-negative (triple-negative) metastatic breast cancer: Results of the randomized phase II bali-1 trial. Cancer Res..

[30] Pavese F., Capoluongo E.D., Muratore M., Minucci A., Santonocito C., Fuso P., Concolino P., Di Stasio E., Carbognin L., Tiberi G. (2022). BRCA Mutation Status in Triple-Negative Breast Cancer Patients Treated with Neoadjuvant Chemotherapy: A Pivotal Role for Treatment Decision-Making. Cancers.

[31] Choi E., Mun G.-I., Lee J., Lee H., Cho J., Lee Y.-S. (2023). BRCA1 deficiency in triple-negative breast cancer: Protein stability as a basis for therapy. Biomed. Pharmacother..

[32] Xu Y., Diao L., Chen Y., Liu Y., Wang C., Ouyang T., Li J., Wang T., Fan Z., Fan T. (2013). Promoter methylation of BRCA1 in triple-negative breast cancer predicts sensitivity to adjuvant chemotherapy. Ann. Oncol..

[33] Yamashita N., Tokunaga E., Kitao H., Hitchins M., Inoue Y., Tanaka K., Hisamatsu Y., Taketani K., Akiyoshi S., Okada S. (2015). Epigenetic inactivation of BRCA1 through promoter hypermethylation and its clinical importance in triple-negative breast cancer. Clin. Breast Cancer.

[34] Zhu X., Shan L., Wang F., Wang J., Wang F., Shen G., Liu X., Wang B., Yuan Y., Ying J. (2015). Hypermethylation of BRCA1 gene: Implication for prognostic biomarker and therapeutic target in sporadic primary triple-negative breast cancer. Breast Cancer Res. Treat..

[35] Veeck J., Ropero S., Setien F., Gonzalez-Suarez E., Osorio A., Benitez J., Herman J.G., Esteller M. (2010). BRCA1 CpG island hypermethylation predicts sensitivity to poly(adenosine diphosphate)-ribose polymerase inhibitors. J. Clin. Oncol..

[36] Glodzik D., Bosch A., Hartman J., Aine M., Vallon-Christersson J., Reutersward C., Karlsson A., Mitra S., Nimeus E., Holm K. (2020). Comprehensive molecular comparison of BRCA1 hypermethylated and BRCA1 mutated triple negative breast cancers. Nat. Commun..

[37] Prajzendanc K., Domagała P., Hybiak J., Ryś J., Huzarski T., Szwiec M., Tomiczek-Szwiec J., Redelbach W., Sejda A., Gronwald J. (2020). BRCA1 promoter methylation in peripheral blood is associated with the risk of triple-negative breast cancer. Int. J. Cancer.

[38] Gardi N., Chaubal R., Parab P., Pachakar S., Kulkarni S., Shet T., Joshi S., Kembhavi Y., Chandrani P., Quist J. (2024). Natural history of germline BRCA1 mutated and BRCA wild-type triple-negative breast cancer. Cancer Res. Commun..

[39] Feng C., Zhang Y., Wu F., Li J., Liu M., Lv W., Li C., Wang W., Tan Q., Xue X. (2023). Relationship between homologous recombination deficiency and clinical features of breast cancer based on genomic scar score. Breast.

[40] Creeden J.F., Nanavaty N.S., Einloth K.R., Gillman C.E., Stanbery L., Hamouda D.M., Dworkin L., Nemunaitis J. (2021). Homologous recombination proficiency in ovarian and breast cancer patients. BMC Cancer.

[41] Chang H.H.Y., Pannunzio N.R., Adachi N., Lieber M.R. (2017). Non-Homologous DNA End Joining and Alternative Pathways to Double-Strand Break Repair. Nat. Rev. Mol. Cell Biol..

[42] Stinson B.M., Loparo J.J. (2021). Repair of DNA Double-Strand Breaks by the Non-homologous End Joining Pathway. Annu. Rev. Biochem..

[43] Fu X., Tan W., Song Q., Pei H., Li J. (2022). BRCA1 and Breast Cancer: Molecular Mechanisms and Therapeutic Strategies. Front. Cell Dev. Biol..

[44] Moreno N.N., Olthof A.M., Svejstrup J.Q. (2023). Transcription-Coupled Nucleotide Excision Repair and the Transcriptional Response to UV-Induced DNA Damage. Annu. Rev. Biochem..

[45] Atipairin A., Ratanaphan A. (2011). In vitro enhanced sensitivity to cisplatin in D67Y BRCA1 RING domain protein. Breast Cancer Basic Clin. Res..

[46] Atipairin A., Canyuk B., Ratanaphan A. (2011). The RING heterodimer BRCA1-BARD1 is a ubiquitin ligase inactivated by the platinum-based anticancer drugs. Breast Cancer Res. Treat..

[47] Nhukeaw T., Temboot P., Hansongnern K., Ratanaphan A. (2014). Cellular responses of BRCA1-defective and triple-negative breast cancer cells and in vitro BRCA1 interactions induced by metallo-intercalator ruthenium(II) complexes containing chloro-substituted phenylazopyridine. BMC Cancer.

[48] Akashi-Tanaka S., Watanabe C., Takamaru T., Kuwayama T., Ikeda M., Ohyama H., Mori M., Yoshida R., Hashimoto R., Terumasa S. (2015). BRCAness Predicts Resistance to Taxane-Containing Regimens in Triple Negative Breast Cancer During Neoadjuvant Chemotherapy. Clin. Breast Cancer.

[49] Isakoff S.J., Mayer E.L., He L., Traina T.A., Carey L.A., Krag K.J., Rugo H.S., Liu M.C., Stearns V., Come S.E. (2015). TBCRC009: A multicenter phase II clinical trial of platinum monotherapy with biomarker assessment in metastatic triple-negative breast cancer. J. Clin. Oncol..

[50] Bernges F., Holler E. (1991). The reaction of platinum(II) complexes with DNA. Kinetics of intrastrand crosslink formation in vitro. Nucleic Acids Res..

[51] Niu Q., Zhang T. (2025). Synergistic mechanism of olaparib and cisplatin on breast cancer elucidated by network pharmacology. Sci. Rep..

[52] Hongthong K., Nhukeaw T., Temboot P., Dyson P.J., Ratanaphan A. (2021). Anticancer activity of RAPTA-EA1 in triple-negative BRCA1 proficient breast cancer cells: Single and combined treatment with the PARP inhibitor olaparib. Heliyon.

[53] Slamon D., Eiermann W., Robert N., Pienkowski T., Martin M., Press M., Mackey J., Glaspy J., Chan A., Pawlicki M. (2011). Adjuvant Trastuzumab in HER2-Positive Breast Cancer. N. Engl. J. Med..

[54] Waks A.G., Winer E.P. (2019). Breast Cancer Treatment: A Review. JAMA.

[55] Isakoff S.J., Goss P.E., Mayer E.L., Traina T.A., Carey L.A., Krag K. (2011). TBCRC009: A multicenter phase II study of cisplatin or carboplatin for metastatic triple-negative breast cancer and evaluation of p63/p73 as a biomarker of response. J. Clin. Oncol..

[56] Tutt A., Robson M., Garber J.E., Domchek S.M., Audeh M.W., Weitzel J.N., Friedlander M., Arun B., Loman N., Schmutzler R.K. (2010). Oral poly(ADP-ribose) polymerase inhibitor olaparib in patients with BRCA1 or BRCA2 mutations and advanced breast cancer: A proof-of-concept trial. Lancet.

[57] Rodler E., Sharma P., Barlow W.E., Gralow J.R., Puhalla S.L., Anders C.K., Goldstein L., Tripathy D., Brown-Glaberman U.A., Huynh T.-T. (2023). Cisplatin with veliparib or placebo in metastatic triple-negative breast cancer and BRCA mutation-associated breast cancer (S1416): A randomised, double-blind, placebo-controlled, phase 2 trial. Lancet Oncol..

[58] Fan Y., Xu B.H., Yuan P., Cai R.G. (2012). P5-19-04: Results of a randomized phase II study demonstrate benefit of platinum-based regimen in the first-line treatment of triple negative breast cancer (TNBC). Cancer Res..

[59] von Minckwitz G., Schneeweiss A., Loibl S., Salat C., Denkert C., Rezai M., Blohmer J.U., Jackisch C., Paepke S., Gerber B. (2014). Neoadjuvant carboplatin in patients with triple-negative and HER2-positive early breast cancer (GeparSixto; GBG 66): A randomised phase 2 trial. Lancet Oncol..

[60] Rugo H.S., Olopade O.I., DeMichele A., Yau C., Veer L.J.V.T., Buxton M.B., Hogarth M., Hylton N.M., Paoloni M., Perlmutter J. (2016). Adaptive Randomization of Veliparib-Carboplatin Treatment in Breast Cancer. N. Engl. J. Med..

[61] Loibl S., O’Shaughnessy J., Untch M., Sikov W.M., Rugo H.S., McKee M.D., Huober J., Golshan M., Von Minckwitz G., Maag D. (2018). Addition of the PARP inhibitor veliparib plus carboplatin or carboplatin alone to standard neoadjuvant chemotherapy in triple-negative breast cancer (BrighTNess): A randomised, phase 3 trial. Lancet Oncol..

[62] O’Shaughnessy J., Schwartzberg L., Danso M.A., Miller K.D., Rugo H.S., Neubauer M., Robert N., Hellerstedt B., Saleh M., Richards P. (2014). Phase III study of iniparib plus gemcitabine and carboplatin versus gemcitabine and carboplatin in patients with metastatic triple-negative breast cancer. J. Clin. Oncol..

[63] Le Tourneau C., Dettwiler S., Beuzboc P., Alran S., Laurence V., Pierga J.Y., Fréneaux P., Sigal-Zafrani B., Diéras V., Vincent-Salomon A. (2012). Pathologic response to short intensified taxane-free neoadjuvant chemotherapy in patients with proliferative operable breast cancer. Am. J. Clin. Oncol..

[64] Sikov W.M., Polley M.-Y., Twohy E., Perou C.M., Singh B., Berry D.A., Tolaney S.M., Somlo G., Port E.R., Ma C.X. (2019). CALGB (Alliance) 40603: Long-term outcomes (LTOs) after neoadjuvant chemotherapy (NACT) +/- carboplatin (Cb) and bevacizumab (Bev) in triple-negative breast cancer (TNBC). J. Clin. Oncol..

[65] Abraham J.E., Pinilla K., Dayimu A., Grybowicz L., Demiris N., Harvey C., Drewett L.M., Lucey R., Fulton A., Roberts A.N. (2024). The PARTNER trial of neoadjuvant olaparib with chemotherapy in triple-negative breast cancer. Nature.

[66] Sirohi B., Arnedos M., Popat S., Ashley S., Nerurkar A., Walsh G., Johnston S., Smith I.E. (2008). Platinum-based chemotherapy in triple-negative breast cancer. Ann. Oncol..

[67] Telli M.L., Jensen K.C., Vinayak S., Kurian A.W., Lipson J.A., Flaherty P.J., Timms K., Abkevich V., Schackmann E.A., Wapnir I.L. (2015). Phase II Study of Gemcitabine, Carboplatin, and Iniparib As Neoadjuvant Therapy for Triple-Negative and BRCA1/2 Mutation-Associated Breast Cancer With Assessment of a Tumor-Based Measure of Genomic Instability: PrECOG 0105. J. Clin. Oncol..

[68] Tung N.M., Robson M.E., Ventz S., Santa-Maria C.A., Nanda R., Marcom P.K., Shah P.D., Ballinger T.J., Yang E.S., Vinayak S. (2020). TBCRC 048: Phase II Study of Olaparib for Metastatic Breast Cancer and Mutations in Homologous Recombination-Related Genes. J. Clin. Oncol..

[69] Li X., Yang J., Peng L., Sahin A.A., Huo L., Ward K.C., O’Regan R., Torres M.A., Meisel J.L. (2017). Triple-negative breast cancer has worse overall survival and cause-specific survival than non-triple-negative breast cancer. Breast Cancer Res. Treat..

[70] Wood R.D., Mitchell M., Sgouros J., Lindahl T. (2001). Human DNA repair genes. Science.

[71] Fasching P.A., Link T., Hauke J., Seither F., Jackisch C., Klare P., Schmatloch S., Hanusch C., Huober J., Stefek A. (2021). Neoadjuvant paclitaxel/olaparib in comparison to paclitaxel/carboplatinum in patients with HER2-negative breast cancer and homologous recombination deficiency (GeparOLA study). Ann. Oncol..

[72] Fasching P.A., Schmatloch S., Hauke J., Rey J., Jackisch C., Klare P., Link T., Hanusch C., Huober J., Stefek A. (2025). Neoadjuvant Paclitaxel/Olaparib in Comparison to Paclitaxel/Carboplatin in Patients with HER2-Negative Breast Cancer and HRD-Long-term Survival of the GeparOLA Study. Clin. Cancer Res..

[73] Farmer H., McCabe N., Lord C.J., Tutt A.N., Johnson D.A., Richardson T.B., Santarosa M., Dillon K.J., Hickson I., Knights C. (2005). Targeting the DNA repair defect in BRCA mutant cells as a therapeutic strategy. Nature.

[74] Caldecott K.W. (2024). Causes and consequences of DNA single-strand breaks. Trends Biochem. Sci..

[75] Zandarashvili L., Langelier M.-F., Velagapudi U.K., Hancock M.A., Steffen J.D., Billur R., Hannan Z.M., Wicks A.J., Krastev D.B., Pettitt S.J. (2020). Structural basis for allosteric PARP-1 retention on DNA breaks. Science.

[76] Cai S.X., Ma N., Wang X., Guo M., Jiang Y., Tian Y.E. (2025). The Discovery of a Potent PARP1 Inhibitor Senaparib. Mol. Cancer Ther..

[77] Rudolph J., Jung K., Luger K. (2022). Inhibitors of PARP: Number crunching and structure gazing. Proc. Natl. Acad. Sci. USA.

[78] Hunia J., Gawalski K., Szredzka A., Suskiewicz M.J., Nowis D. (2022). The potential of PARP inhibitors in targeted cancer therapy and immunotherapy. Front. Mol. Biosci..

[79] Loap P., Loirat D., Berger F., Ricci F., Vincent-Salomon A., Ezzili C., Mosseri V., Fourquet A., Ezzalfani M., Kirova Y. (2021). Combination of Olaparib and Radiation Therapy for Triple Negative Breast Cancer: Preliminary Results of the Radioparp Phase 1 Trial. Int. J. Radiat. Oncol. Biol. Phys..

[80] Taylor A.K., Kosoff D., Emamekhoo H., Lang J.M., Kyriakopoulos C.E. (2023). PARP inhibitors in metastatic prostate cancer. Front. Oncol..

[81] Rouleau-Turcotte E., Pascal J.M. (2023). ADP-ribose contributions to genome stability and PARP enzyme trapping on sites of DNA damage; paradigm shifts for a coming-of-age modification. J. Biol. Chem..

[82] Mateo J., Lord C.J., Serra V., Tutt A., Balmanña J., Castroviejo-Bermejo M., Cruz C., Oaknin A., Kaye S.B., de Bono J.S. (2019). A decade of clinical development of PARP inhibitors in perspective. Ann. Oncol..

[83] Habaka M., Daly G.R., Shinyanbola D., Alabdulrahman M., McGrath J., Dowling G.P., Hehir C., Huang H.Y.R., Hill A.D.K., Varešlija D. (2025). PARP Inhibitors in the Neoadjuvant Setting; A Comprehensive Overview of the Rationale for their Use, Past and Ongoing Clinical Trials. Curr. Oncol. Rep..

[84] Li Q., Qian W., Zhang Y., Hu L., Chen S., Xia Y. (2023). A new wave of innovations within the DNA damage response. Sig. Transduct. Target Ther..

[85] Zeng Y., Arisa O., Peer C.J., Fojo A., Figg W.D. (2024). PARP inhibitors: A review of the pharmacology, pharmacokinetics, and pharmacogenetics. Semin. Oncol..

[86] Xiong Y., Guo Y., Liu Y., Wang H., Gong W., Liu Y., Wang X., Gao Y., Yu F., Su D. (2020). Pamiparib is a potent and selective PARP inhibitor with unique potential for the treatment of brain tumor. Neoplasia.

[87] Herencia-Ropero A., Llop-Guevara A., Staniszewska A.D., Domènech-Vivó J., García-Galea E., Moles-Fernández A., Pedretti F., Domènech H., Rodríguez O., Guzmán M. (2024). The PARP1 selective inhibitor saruparib (AZD5305) elicits potent and durable antitumor activity in patient-derived BRCA1/2-associated cancer models. Genome Med..

[88] Hobbs E.A., Litton J.K., Yap T.A. (2021). Development of the PARP inhibitor talazoparib for the treatment of advanced BRCA1 and BRCA2 mutated breast cancer. Expert Opin. Pharmacother..

[89] Gruber J.J., Afghahi A., Timms K., DeWees A., Gross W., Aushev V.N., Wu H.-T., Balcioglu M., Sethi H., Scott D. (2022). A Phase II Study of Talazoparib Monotherapy in Patients with Wild-Type Brca1 and Brca2 with a Mutation in Other Homologous Recombination Genes. Nat. Cancer.

[90] Litton J.K., Hurvitz S.A., Mina L.A., Rugo H.S., Lee K.-H., Gonçalves A., Diab S., Woodward N., Goodwin A., Yerushalmi R. (2020). Talazoparib versus chemotherapy in patients with germline BRCA1/2-mutated HER2-negative advanced breast cancer: Final overall survival results from the EMBRACA trial. Ann. Oncol..

[91] Xu B., Sun T., Shi Y., Cui J., Yin Y., Ouyang Q., Liu Q., Zhang Q., Chen Y., Wang S. (2023). Pamiparib in patients with locally advanced or metastatic HER2-negative breast cancer with germline BRCA mutations: A phase II study. Breast Cancer Res. Treat..

[92] Groelly F.J., Fawkes M., Dagg R.A., Blackford A.N., Tarsounas M. (2023). Targeting DNA Damage Response Pathways in Cancer. Nat. Rev. Cancer.

[93] Krastev D.B., Li S., Sun Y., Wicks A.J., Hoslett G., Weekes D., Badder L.M., Knight E.G., Marlow R., Pardo M.C. (2022). The Ubiquitin-Dependent ATPase P97 Removes Cytotoxic Trapped Parp1 from Chromatin. Nat. Cell Biol..

[94] Zatreanu D., Robinson H.M.R., Alkhatib O., Boursier M., Finch H., Geo L., Grande D., Grinkevich V., Heald R.A., Langdon S. (2021). Poltheta Inhibitors Elicit BRCA-Gene Synthetic Lethality and Target PARP Inhibitor Resistance. Nat. Commun..

[95] Zou Y., Zhang H., Chen P., Tang J., Yang S., Nicot C., Guan Z., Li X., Tang H. (2025). Clinical approaches to overcome PARP inhibitor resistance. Mol. Cancer.

[96] Zhang J., Huang Z., Song C., Wu S., Xie J., Zou Y., Xie X., Wu T., Yang H., Tang H. (2025). PRMT1-Mediated PARP1 Methylation Drives Lung Metastasis and Chemoresistance via P65 Activation in Triple-Negative Breast Cancer. Research.

[97] Eckstein N. (2011). Platinum resistance in breast and ovarian cancer cell lines. J. Exp. Clin. Cancer Res..

[98] Martin L.P., Hamilton T.C., Schilder R.J. (2008). Platinum resistance: The role of DNA repair pathways. Clin. Cancer Res..

[99] Kartalou M., Essigmann J.M. (2001). Recognition of cisplatin adducts by cellular proteins. Mutat. Res..

[100] Gately D.P., Howell S.B. (1993). Cellular accumulation of the anticancer agent cisplatin: A review. Br. J. Cancer.

[101] Kuo Y.M., Zhou B., Cosco D., Gitschier J. (2001). The copper transporter CTR1 provides an essential function in mammalian embryonic development. Proc. Natl. Acad. Sci. USA.

[102] Howell S.B., Safaei R., Larson C.A., Sailor M.J. (2010). Copper transporters and the cellular pharmacology of the platinum-containing cancer drugs. Mol. Pharmacol..

[103] Kuo M.T., Fu S., Savaraj N., Chen H.H. (2012). Role of the human high-affinity copper transporter in copper homeostasis regulation and cisplatin sensitivity in cancer chemotherapy. Cancer Res..

[104] Katano K., Safaei R., Samimi G., Holzer A., Rochdi M., Howell S.B. (2003). The copper export pump ATP7B modulates the cellular pharmacology of carboplatin in ovarian carcinoma cells. Mol. Pharmacol..

[105] Maxfield A.B., Heaton D.N., Winge D.R. (2004). Cox17 is functional when tethered to the mitochondrial inner membrane. J. Biol. Chem..

[106] Zhao L., Cheng Q., Wang Z., Xi Z., Xu D., Liu Y. (2014). Cisplatin binds to human copper chaperone Cox17: The mechanistic implication of drug delivery to mitochondria. Chem. Commun..

[107] Godwin A.K., Meister A., O’Dwyer P.J., Huang C.S., Hamilton T.C., Anderson M.E. (1992). High resistance to cisplatin in human ovarian cancer cell lines is associated with marked increase of glutathione synthesis. Proc. Natl. Acad. Sci. USA.

[108] Ishikawa T., Ali-Osman F. (1993). Glutathione-associated cis-diamminedichloroplatinum(II) metabolism and ATP-dependent efflux from leukemia cells. Molecular characterization of glutathione-platinum complex and its biological significance. J. Biol. Chem..

[109] Galluzzi L., Senovilla L., Vitale I., Michels J., Martins I., Kepp O., Castedo M., Kroemeret G. (2012). Molecular mechanisms of cisplatin resistance. Oncogene.

[110] Helleday T., Petermann E., Lundin C., Hodgson B., Sharma R.A. (2008). DNA repair pathways as targets for cancer therapy. Nat. Rev. Cancer.

[111] Chabner B.A., Roberts T.G. (2005). Chemotherapy and the war on cancer. Nat. Rev. Cancer.

[112] Sharma R.A., Dianov G.L. (2007). Targeting base excision repair to improve cancer therapies. Mol. Aspects Med..

[113] Sugasawa K., Okamoto T., Shimizu Y., Masutani C., Iwai S., Hanaoka F. (2001). A multistep damage recognition mechanism for global genomic nucleotide excision repair. Gene Dev..

[114] Rabik C.A., Dolan M.E. (2007). Molecular mechanisms of resistance and toxicity associated with platinating agents. Cancer Treat. Rev..

[115] Husain A., He G., Venkatraman E.S., Spriggs D.R. (1998). BRCA1 upregulation is associated with repair-mediated resistance to *cis*-diamminedichloroplatinum(II). Cancer Res..

[116] Quinn J.E., Kennedy R.E., Mullan P.B., Gilmore P.M., Carty M., Johnston P.G., Harkinet D.P. (2003). BRCA1 functions as a differential modulator of chemotherapy-induced apoptosis. Cancer Res..

[117] Swisher E.M., Sakai W., Karlan B.Y., Wurz K., Urban N., Taniguchi T. (2008). Secondary BRCA1 mutations in BRCA1-mutated ovarian carcinomas with platinum resistance. Cancer Res..

[118] Sklar M.D. (1988). Increased resistance to *cis*-diamminedichloroplatinum(II) in NIH 3T3 cells transformed by *ras* oncogenes. Cancer Res..

[119] O’Byrne K.J., Barr M.P., Gray S.G. (2011). The role of epigenetics in resistance to cisplatin chemotherapy in lung cancer. Cancer.

[120] Menghi F., Banda K., Kumar P., Straub R., Dobrolecki L., Rodriguez I.V., Yost S.E., Chandok H., Radke M.R., Somlo G. (2022). Genomic and Epigenomic BRCA Alterations Predict Adaptive Resistance and Response to Platinum-Based Therapy in Patients with Triple-Negative Breast and Ovarian Carcinomas. Sci. Transl. Med..

[121] Menghi F., Banda K., Kumar P., Straub R., Dobrolecki L.E., Rodriguez I., Yost S.E., Chandok H., Radke M., Somlo G. (2023). Abstract PD5-01: PD5-01 Genomic and epigenomic BRCA alterations predict adaptive resistance and response to platinum-based therapy in triple negative breast cancer and ovarian carcinoma. Cancer Res..

[122] Ma X., Cheng Z., Guo C. (2025). Insights into the DNA damage response and tumor drug resistance. Cancer Biol. Med..

[123] Scanlon K.J., Kashani-Sabet M., Miyachi H., Sowers L.C., Rossi J.J. (1989). Molecular basis of cisplatin resistance in human carcinomas: Model systems and patients. Anticancer Res..

[124] Dhar R., Basu A. (2008). Costitutive activation of p70 S6 kinase is associated with intrinsic resistance to cisplatin. Int. J. Oncol..

[125] Aebi S., Kurdi-Haidar B., Gordon R., Cenni B., Zheng H., Fink D., Christen R.D., Boland C.R., Koi M., Fishel R. (1996). Loss of DNA mismatch repair in acquired resistance to cisplatin. Cancer Res..

[126] Hicks J.K., Chute C.L., Paulsen M.T., Ragland R.L., Howlett N.G., Gueranger Q., Glover T.W., Canmanet C.E. (2010). Differential roles for DNA polymerases eta, zeta, and REV1 in lesion bypass of intrastrand versus interstrand DNA cross-links. Mol. Cell. Biol..

[127] Lin K.K., Harrell M.I., Oza A.M., Oaknin A., Ray-Coquard I., Tinker A.V., Helman E., Radke M.R., Say C., Vo L.T. (2019). BRCA Reversion Mutations in Circulating Tumor DNA Predict Primary and Acquired Resistance to the PARP Inhibitor Rucaparib in High-Grade Ovarian Carcinoma. Cancer Discov..

[128] Patel A.G., Sarkaria J.N., Kaufmann S.H. (2011). Nonhomologous end joining drives poly(ADP-ribose) polymerase (PARP) inhibitor lethality in homologous recombination-deficient cells. Proc. Natl. Acad. Sci. USA.

[129] Rondinelli B., Gogola E., Yucel H., Duarte A.A., van de Ven M., van der Sluijs R., Konstantinopoulos P.A., Jonkers J., Ceccaldi R., Rottenberg S. (2017). EZH2 promotes degradation of stalled replication forks by recruiting MUS81 through histone H3 trimethylation. Nat. Cell Biol..

[130] Taglialatela A., Alvarez S., Leuzzi G., Sannino V., Ranjha L., Huang J.W., Madubata C., Anand R., Levy B., Rabadan R. (2017). Restoration of Replication Fork Stability in BRCA1- and BRCA2-Deficient Cells by Inactivation of SNF2-Family Fork Remodelers. Mol. Cell.

[131] Matsumoto K., Matsumoto Y., Wada J. (2025). PARylation-mediated post-transcriptional modifications in cancer immunity and immunotherapy. Front. Immunol..

[132] Noordermeer S.M., van Attikum H. (2019). PARP Inhibitor Resistance: A Tug-of-War in BRCA-Mutated Cells. Trends Cell Biol..

[133] Carnero A., Blanco-Aparicio C., Renner O., Link W., Leal J.F. (2008). The PTEN/PI3K/AKT signalling pathway in cancer, therapeutic implications. Curr. Cancer Drug Targets.

[134] Saal L.H., Gruvberger-Saal S.K., Persson C., Lovgren K., Jumppanen M., Staaf J., Jonsson G., Pires M.M., Maurer M., Holm K. (2008). Recurrent gross mutations of the PTEN tumor suppressor gene in breast cancers with deficient DSB repair. Nat. Genet..

[135] Mendes-Pereira A.M., Martin S.A., Brough R., McCarthy A., Taylor J.R., Kim J.S., Waldman T., Lord C.J., Ashworth A. (2009). Synthetic lethal targeting of PTEN mutant cells with PARP inhibitors. EMBO Mol. Med..

[136] Piombino C., Cortesi L. (2022). Insights into the Possible Molecular Mechanisms of Resistance to PARP Inhibitors. Cancers.

[137] Choi Y.H., Yu A.M. (2014). ABC transporters in multidrug resistance and pharmacokinetics, and strategies for drug development. Curr. Pharm. Des..

[138] Rottenberg S., Jaspers J.E., Kersbergen A., van der Burg E., Nygren A.O., Zander S.A., Derksen P.W., de Bruin M., Zevenhoven J., Lau A. (2008). High sensitivity of BRCA1-deficient mammary tumors to the PARP inhibitor AZD2281 alone and in combination with platinum drugs. Proc. Natl. Acad. Sci. USA.

[139] Leitner I., Nemeth J., Feurstein T., Abrahim A., Matzneller P., Lagler H., Erker T., Langer O., Zeitlinger M. (2011). The third-generation P-glycoprotein inhibitor tariquidar may overcome bacterial multidrug resistance by increasing intracellular drug concentration. J. Antimicrob. Chemother..

[140] Christie E.L., Pattnaik S., Beach S.J., Copeland A., Rashoo N., Fereday S., Hendley J., Alsop K., Brady S.L., Lamb G. (2019). Multiple ABCB1 transcriptional fusions in drug resistant high-grade serous ovarian and breast cancer. Nat. Commun..

[141] Choi Y.E., Meghani K., Brault M.-E., Leclerc L., He Y.J., Day T.A., Elias K.M., Drapkin R., Weinstock D.M., Dao F. (2016). Platinum and PARP Inhibitor Resistance Due to Overexpression of MicroRNA-622 in BRCA1-Mutant Ovarian Cancer. Cell Rep..

[142] Wang Y., Krais J.J., Bernhardy A.J., Nicolas E., Cai K.Q., Harrell M.I., Kim H.H., George E., Swisher E.M., Simpkins F. (2016). RING domain-deficient BRCA1 promotes PARP inhibitor and platinum resistance. J. Clin. Investig..

[143] Dias M.P., Moser S.C., Ganesan S., Jonkers J. (2021). Understanding and Overcoming Resistance to PARP Inhibitors in Cancer Therapy. Nat. Rev. Clin. Oncol..

[144] Morganti S., Marra A., De Angelis C., Toss A., Licata L., Giugliano F., Salimbeni B.T., Giachetti P.P.M.B., Esposito A., Giordano A. (2024). PARP Inhibitors for Breast Cancer Treatment: A Review. JAMA Oncol..

[145] Sargazi S., Mukhtar M., Rahdar A., Barani M., Pandey S., Diez-Pascual A.M. (2021). Active targeted nanoparticles for delivery of poly(ADP-ribose) polymerase (PARP) inhibitors: A preliminary review. Int. J. Mol. Sci..

[146] Fu Z., Li S., Han S., Shi C., Zhang Y. (2022). Antibody drug conjugate: The “biological missile” for targeted cancer therapy. Sig. Transduct. Target Ther..

[147] Schluga P., Hartinger C.G., Egger A., Reisner E., Galanski M., Jakupec M.A., Keppler B.K. (2006). Redox behavior of tumor-inhibiting ruthenium(III) complexes and effects of physiological reductants on their binding to GMP. Dalton Trans..

[148] Jakupec M.A., Reisner E., Eichinger A., Pongratz M., Arion V.B., Galanski M., Hartinger C.G., Keppler B.K. (2005). Redox-active antineoplastic ruthenium complexes with indazole: Correlation of in vitro potency and reduction potential. J. Med. Chem..

[149] Ang W.H., Dyson P.J. (2006). Classical and non-classical ruthenium-based anticancer drugs: Towards targeted chemotherapy. Eur. J. Inorg. Chem..

[150] Hartinger C.G., Zorbas-Seifried S., Jakupec M.A., Kynast B., Zorbas H., Keppler B.K. (2006). From bench to bedside-preclinical and early clinical development of the anticancer agent indazolium trans-[tetrachlorobis(1H-indazole)ruthenate(III)] (KP1019 or FFC14A). J. Inorg. Biochem..

[151] Hartinger C.G., Jakupec M.A., Zorbas-Seifried S., Groessl M., Egger A., Berger W., Zorbas H., Dyson P.J., Keppler B.K. (2008). KP1019, a new redox-active anticancer agent--preclinical development and results of a clinical phase I study in tumor patients. Chem. Biodivers..

[152] Clarke M.J. (2003). Ruthenium metallopharmaceuticals. Coord. Chem. Rev..

[153] Sulyok J.M., Hann S., Hartinger C.G., Keppler B.K., Stingeder G., Koellensperger G. (2005). Two dimensional separation schemes for investigation of the interaction of an anticancer ruthenium (III) compound with plasma proteins. J. Anal. At. Spectrom..

[154] Ratanaphan A., Temboot P., Dyson P.J. (2010). In vitro Ruthenation of Human Breast Cancer Suppressor Gene 1 (BRCA1) by the Antimetastasis Compound RAPTA-C and Its Analogue CarboRAPTA-C. Chem. Biodivers..

[155] Ang W.H., Casini A., Sava G., Dyson P.J. (2011). Organometallic ruthenium-based antitumor compounds with novel modes of action. J. Org. Chem..

[156] Chakree K., Ovatlarnporn C., Dyson P.J., Ratanaphan A. (2012). Altered DNA binding and amplification of human breast cancer suppressor gene BRCA1 induced by a novel antitumor compound, [Ru(η^6^-*p*-phenylethacrynate)Cl_2_(pta)]. Int. J. Mol. Sci..

[157] Chatterjee S., Kundu S., Bhattacharyya A., Hartinger C.G., Dyson P.J. (2008). The ruthenium(II)-arene compound RAPTA-C induces apoptosis in EAC cells through mitochondrial and p53-JNK pathways. J. Biol. Inorg. Chem..

[158] Klaimanee E., Temram T., Ratanaphan A., Saithong S., Sooksawat D., Samphao A., Yakiyama Y., Sakurai H., Konno T., Tantirungrotechai Y. (2025). Iridium(III) coordination compounds based on organophosphorus ancillary ligands showing cytotoxicity against breast cancer cells and Fe(III) luminescent sensing. Spectrochim. Acta Part A Mol. Biomol. Spectrosc..

[159] Sojka M., Gamez P. (2025). Exploring the toxicity of mononuclear piano-stool Ru(II) anticancer agents: A comprehensive literature review. Coord. Chem. Rev..

[160] Temram T., Klaimanee E., Saithong S., Amornpitoksuk P., Phongpaichit S., Ratanaphan A., Tantirungrotechai Y., Leesakul N. (2023). Iridium(III) complexes based on cyanomethane and cyanamide ligands with luminescence quenching properties for Fe(III) sensing and biological activities. Polyhedron.

[161] Leesakul N., Kullawanichaiyanan K., Mutić S., Guzsvány V., Nhukeaw T., Ratanaphan A., Saithong S., Konno T., Sirimahachai U., Promarak V. (2021). A photoactive iridium(III) complex with 3-methyl-2-phenyl pyridine and 1,1-bis(diphenylphosphino)methane: Synthesis, structural characterization and cytotoxicity in breast cancer cells. J. Coord. Chem..

[162] Bibi R., Zahid M., Rasool F., Tariq M., Hussain A., Asif H.M., Khan M.A., Shah K.H., Hussain S., Sirajuddin M. (2025). Synthesis, spectroscopic, computational, molecular docking, antidiabetic (in vitro & in vivo) DNA and BSA interaction studies of ruthenium(II) carboxylate complexes. Spectrochim. Acta Part A Mol. Biomol. Spectrosc..

[163] Kim W.K., An J.M., Lim Y.J., Kim K., Kim Y.H., Kim D. (2025). Recent advances in metallodrug: Coordination-induced synergy between clinically approved drugs and metal ions. Mater. Today Adv..

[164] Kozieł S., Wojtala D., Szmitka M., Lesiów M., Ziółkowska A., Sawka J., Carpio E.D., Crans D.C., Komarnicka U.K. (2025). Half-Sandwich Organometallic Ir(III) and Ru(II) Compounds and their Interactions with Biomolecules. ChemPlusChem.

[165] Mandal A. (2024). Alternative of cisplatin—Introduction of rhodium analogues. J. Indian Chem. Soc..

[166] Bashir M., Mantoo I.A., Prasad C.P., Yousuf I. (2024). New mono and dinuclear half-sandwiched organoruthenium(II) complexes: Effect of dinuclearity on the biomolecular interaction and cytotoxicity. Inorg. Chim. Acta.

[167] Klaimanee E., Nhukeaw T., Saithong S., Ratanaphan A., Phongpaichit S., Tantirungrotechai Y., Leesakul N. (2021). Half-sandwich ruthenium (II) p-cymene complexes based on organophosphorus ligands: Structure determination, computational investigation, in vitro antiproliferative effect in breast cancer cells and antimicrobial activity. Polyhedron.

[168] Bhattacharya S., Adon T., Dsouza K., Kumar H.Y. (2025). Exploring the Future of Metal-Based Anticancer Agents: A Comprehensive Review of Ruthenium-Based Complexes. ChemistrySelect.

[169] Sahu G., Patra S.A., Lima S., Das S., Görls H., Plass W., Dinda R. (2023). Ruthenium(II)-Dithiocarbazates as Anticancer Agents: Synthesis, Solution Behavior, and Mitochondria-Targeted Apoptotic Cell Death. Chem. Eur. J..

[170] Katheria S. (2022). Ruthenium Complexes as Potential Cancer Cell Growth Inhibitors for Targeted Chemotherapy. ChemistrySelect.

[171] Scolaro C., Bergamo A., Brescacin L., Delfino R., Cocchietto M., Laurenczy G., Geldbach T.J., Sava G., Dyson P.J. (2005). In Vitro and in Vivo Evaluation of Ruthenium(II)-Arene PTA Complexes. J. Med. Chem..

[172] Lorenzon T., Vescovo M., Maiullari M., Tonon G., Conceiçao N.R., Carabineiro S.A.C., Mahmoud A.G., Dietl M.C., Demitri N., Orian L. (2025). Influence of the charge of 1,3,5-triaza-7-phosphaadamantane-based ligands on the anticancer activity of organopalladium complexes. RSC Adv..

[173] Nhukeaw T., Hongthong K., Dyson P.J., Ratanaphan A. (2019). Cellular responses of *BRCA1*-defective HCC1937 breast cancer cells induced by the antimetastasis ruthenium(II) arene compound RAPTA-T. Apoptosis.

[174] Babak M.V., Meier S.M., Huber K., Reynisson J., Legin A.A., Jakupec M., Roller A., Stukalov A., Gridling M., Bennett K.L. (2015). Target profiling of an antimetastatic RAPTA agent by chemical proteomics: Relevance to the mode of action. Chem. Sci..

[175] Ang W.H., Parker L.J., De Luca A., Juillerat-Jeanneret L., Morton C.J., Bello M.L., Parker M.W., Dyson P.J. (2009). Rational design of an organometallic glutathione transferase inhibitor. Angew. Chem. Int. Ed. Engl..

[176] Bernal G., Aquea G., Ramírez-Rivera S. (2025). Metal-based molecules in the treatment of cancer: From bench to bedside. Oncol. Res..

[177] Amato A.D., Mariconda A., Iacopetta D., Ceramella J., Catalano A., Sinicropi M.S., Longo P. (2023). Complexes of Ruthenium(II) as Promising Dual-Active Agents against Cancer and Viral Infections. Pharmaceuticals.

[178] Heric A., Dibranin N., Martic L., Hodzic E., Zahirovic A., Kozaric A.K. (2024). Ruthenium-based complexes as antitumor agents. J. Health Sci..

[179] Levina A., Chetcuti A.R.M., Lay P.A. (2022). Controversial role of transferrin in the transport of ruthenium anticancer drugs. Biomolecules.

[180] Nayeem N., Sauma S., Ahad A., Rameau R., Kebadze S., Bazett M., Park B.J., Casaccia P., Prabha S., Hubbard K. (2024). Insights into mechanisms and promising triple negative breast cancer therapeutic potential for a water-soluble ruthenium compound. ACS Pharmacol. Transl. Sci..

[181] Adhireksan Z., Davey G.E., Campomanes P., Groessl M., Clavel C.M., Yu H., Nazarov A.A., Yeo C.H.F., Ang W.H., Dröge P. (2014). Ligand substitutions between ruthenium-cymene compounds can control protein versus DNA targeting and anticancer activity. Nat. Commun..

[182] Wu B., Ong M.S., Groessl M., Adhireksan Z., Hartinger C.G., Dyson P.J., Davey C.A. (2011). A ruthenium antimetastasis agent forms specific histone protein adducts in the nucleosome core. Chem. Eur. J..

[183] Adhireksan Z., Palermo G., Riedel T., Ma Z., Muhammad R., Rothlisberger U., Dyson P.J., Davey C.A. (2017). Allosteric crosstalk in chromatin can mediate drug-drug synergy. Nat. Commun..

[184] Berndsen R.H., Weiss A., Abdul U.K., Wong T.J., Meraldi P., Griffioen A.W., Dyson P.J., Nowak-Sliwinska P. (2017). Combination of ruthenium(II)-arene complex [Ru(*η*^6^-*p*-cymene)Cl_2_(pta)] (RAPTA-C) and the epidermal growth factor receptor inhibitor erlotinib results in efficient angiostatic and antitumor activity. Sci. Rep..

[185] Swaminathan S., Karvembu R. (2023). Dichloro Ru(II)-p-cymene-1,3,5-triaza-7-phosphaadamantane(RAPTA-C): A Case Study. ACS Pharmacol. Transl. Sci..

[186] Romagnolo A.P.G., Romagnolo D.F., Selmin O. (2015). BRCA1 as Target for Breast Cancer Prevention and Therapy. Anti-Cancer Agents Med. Chem..

[187] Hongthong K., Ratanaphan A. (2016). *BRCA1*-associated triple-negative breast cancer and potential treatment for ruthenium-based compounds. Curr. Cancer Drug Targets.

[188] Ratanaphan A., Nhukeaw T., Hongthong K., Dyson P.J. (2017). Differential Cytotoxicity, Cellular Uptake, Apoptosis and Inhibition of BRCA1 Expression of BRCA1-Defective and Sporadic Breast Cancer Cells Induced by an Anticancer Ruthenium (II)-Arene Compound, RAPTA-EA1. Anti-Cancer Agents Med. Chem..

[189] Temboot P., Lee R.F.S., Menin L., Dyson P.J., Ratanaphan A. (2017). Biochemical and biophysical characterization of ruthenation of BRCA1 RING protein by RAPTA complexes and its E3 ubiquitin ligase activity. Biochem. Biophys. Res. Commun..

[190] Toss A., Cristofanilli M. (2015). Molecular characterization and targeted therapeutic approaches in breast cancer. Breast Cancer Res..

[191] Carvalho E., Canberk S., Schmitt F., Vale N. (2025). Molecular Subtypes and Mechanisms of Breast Cancer: Precision Medicine Approaches for Targeted Therapies. Cancers.

[192] Yusoh N.A., Ahmad H., Gill M.R. (2020). Combining PARP Inhibition with Platinum, Ruthenium or Gold Complexes for Cancer Therapy. ChemMedChem.

[193] Pilie P., Gay C.M., Byers L.A., O’Connor M.J., Yap T.A. (2019). PARP inhibitors: Extending benefit beyond BRCA mutant cancers. Clin. Cancer Res..

[194] Thein K.Z., Thawani R., Kummar S. (2023). Combining Poly (ADP-Ribose) Polymerase (PARP) Inhibitors with Chemotherapeutic Agents: Promise and Challenges. Cancer Treat Res..

[195] Balmaña J., Tung N.M., Isakoff S.J., Graña B., Ryan P.D., Saura C., Lowe E.S., Frewer P., Winer E., Baselga J. (2014). Phase I trial of olaparib in combination with cisplatin for the treatment of patients with advanced breast, ovarian and other solid tumors. Ann. Oncol..

[196] Bouwman P., Jonkers J. (2014). Molecular pathways: How can *BRCA*-mutated tumors become resistant to PARP inhibitors?. Clin. Cancer Res..

[197] Pan J.-N., Lei L., Ye W.-W., Wang X.-J., Cao W.-M. (2021). BRCA1 Reversion Mutation Confers Resistance to Olaparib and Camrelizumab in a Patient with Breast Cancer Liver Metastasis. J. Breast Cancer.

[198] Waks A.G., Cohen O., Kochupurakkal B., Kim D., Dunn C.E., Buendia J.B., Wander S., Helvie K., Lloyd M.R., Marini L. (2020). Reversion and non-reversion mechanisms of resistance to PARP inhibitor or platinum chemotherapy in BRCA1/2-mutant metastatic breast cancer. Ann. Oncol..

[199] Yusoh N.A., Leong S.W., Chia S.L., Harun S.N., Abdul Rahman M.B., Vallis K.A., Gill M.R., Ahmad H. (2020). Metallointercalator [Ru(dppz)_2_(PIP)]^2+^ Renders BRCA Wild-Type Triple-Negative Breast Cancer Cells Hypersensitive to PARP Inhibition. ACS Chem. Biol..

[200] Fong P.C., Yap T.A., Boss D.S., Carden C.P., Mergui-Roelvink M., Gourley C., De Greve J., Lubinski J., Shanley S., Messiou C. (2010). Poly(ADP)-ribose polymerase inhibition: Frequent durable responses in BRCA carrier ovarian cancer correlating with platinum-free interval. J. Clin. Oncol..

[201] Pandya K., Scher A., Omene C., Ganesan S., Kumar S., Ohri N., Potdevin L., Haffty B., Toppmeyer D.L., George M.A. (2023). Clinical efficacy of PARP inhibitors in breast cancer. Breast Cancer Res. Treat..

[202] Zhang C., Xu C., Gao X., Yao Q. (2022). Platinum-based drugs for cancer therapy and anti-tumor strategies. Theranostics.

[203] Bai Y., Aodeng G., Ga L., Hai W., Ai J. (2023). Research progress of metal anticancer drugs. Pharmaceutics.

[204] Pandy J.G.P., Balolong-Garcia J.C., Valerie M., Ordinario B.C., Victoria F., Que F. (2019). Triple negative breast cancer and platinum-based systemic treatment: A meta-analysis and systematic review. BMC Cancer.

[205] Singh D.D., Parveen A., Yadav D.K. (2021). Role of PARP in TNBC: Mechanism of inhibition, clinical applications, and resistance. Biomedicines.

[206] Golbaghi G., Castonguay A. (2020). Rationally designed ruthenium complexes for breast cancer therapy. Molecules.

[207] Ji S., Chen L., Yu Y., Chen X., Wei L., Gou L., Shi C., Zhuang S. (2025). A comprehensive comparison of PARP inhibitors as maintenance therapy in platinum-sensitive recurrent ovarian cancer: A systematic review and network meta-analysis. J. Ovarian Res..

[208] Yusoh N.A., Ahmad H., Vallis K.A., Gill M.R. (2025). Advances in platinum-based cancer therapy: Overcoming platinum resistance through rational combinatorial strategies. Med. Oncol..

[209] Johnstone T.C., Suntharalingam K., Lippard S.J. (2016). The next generation of platinum drugs: Targeted Pt(II) agents, nanoparticle delivery, and Pt(IV) prodrugs. Chem. Rev..

[210] Kim D., Nam H.J. (2022). PARP inhibitors: Clinical limitations and recent attempts to overcome them. Int. J. Mol. Sci..

[211] Lin Z., Wang L., Xing Z., Wang F., Cheng X. (2024). Update on combination strategies of PARP inhibitors. Cancer Control.

[212] Zhou T., Zhang J. (2025). Therapeutic advances and application of PARP inhibitors in breast cancer. Transl. Oncol..

[213] Rose M., Burgess J.T., O’Byrne K., Richard D.J., Bolderson E. (2020). PARP inhibitors: Clinical relevance, mechanisms of action and tumor resistance. Front. Cell Dev. Biol..

[214] Gonzalez-Angulo A.M., Litton J.K., Broglio K.R., Meric-Bernstam F., Rakkhit R., Cardoso F., Peintinger F., Hanrahan E.O., Sahin A., Guray M. (2009). High risk of recurrence for patients with breast cancer who have human epidermal growth factor receptor 2-positive, node-negative tumors 1 cm or smaller. J. Clin. Oncol..

[215] Lu Y., Zhu D., Le Q., Wang Y., Wang W. (2022). Ruthenium-based antitumor drugs and delivery systems from monotherapy to combination therapy. Nanoscale.

[216] Lee S.Y., Kim C.Y., Nam T.G. (2022). Ruthenium Complexes as Anticancer Agents: A Brief History and Perspectives. Drug Des. Dev. Ther..

[217] Dilmac S., Ozpolat B. (2023). Mechanisms of PARP-Inhibitor-Resistance in BRCA-Mutated Breast Cancer and New Therapeutic Approaches. Cancers.

[218] Jain A., Barge A., Parris C.N. (2025). Combination strategies with PARP inhibitors in BRCA-mutated triple-negative breast cancer: Overcoming resistance mechanisms. Oncogene.

[219] Han Y., Li C.W., Hsu J.M., Hsu J.L., Chan L.C., Tan X., He G.J. (2019). Metformin reverses PARP inhibitors-induced epithelial-mesenchymal transition and PD-L1 upregulation in triple-negative breast cancer. Am. J. Cancer Res..

[220] Quereda V., Bayle S., Vena F., Frydman S.M., Monastyrskyi A., Roush W.R., Duckett D.R. (2019). Therapeutic Targeting of CDK12/CDK13 in Triple-Negative Breast Cancer. Cancer Cell.

[221] Do K.T., Kochupurakkal B., Kelland S., de Jonge A., Hedglin J., Powers A., Quinn N., Gannon C., Vuong L., Parmar K. (2021). Phase 1 Combination Study of the CHK1 Inhibitor Prexasertib and the PARP Inhibitor Olaparib in High-grade Serous Ovarian Cancer and Other Solid Tumors. Clin. Cancer Res..

[222] Valiente C.M., Amir E. (2023). Combining PARP inhibitors and platinum-based chemotherapy in metastatic triple negative and/or BRCA-associated breast cancer. Transl. Cancer Res..

[223] Barchiesi G., Roberto M., Verrico M., Vici P., Tomao S., Tomao F. (2021). Emerging Role of PARP Inhibitors in Metastatic Triple Negative Breast Cancer. Current Scenario and Future Perspectives. Front. Oncol..

[224] Srivastava N., Usmani S.S., Subbarayan R., Saini R., Pandey P.K. (2023). Hypoxia: Syndicating triple negative breast cancer against various therapeutic regimens. Front. Oncol..

